# Evoked Weibel‐Palade Body Exocytosis Modifies the Endothelial Cell Surface by Releasing a Substrate‐Selective Phosphodiesterase

**DOI:** 10.1002/advs.202306624

**Published:** 2024-02-15

**Authors:** Johannes Naß, Julian Terglane, Dagmar Zeuschner, Volker Gerke

**Affiliations:** ^1^ Institute of Medical Biochemistry, Center for Molecular Biology of Inflammation University of Muenster von‐Esmarch‐Str. 56 48149 Muenster Germany; ^2^ Electron Microscopy Facility Max Planck Institute for Molecular Biomedicine Roentgenstr. 20 48149 Muenster Germany

**Keywords:** calcium, complement, GPI anchor, inflammation, secretion

## Abstract

Weibel Palade bodies (WPB) are lysosome‐related secretory organelles of endothelial cells. Commonly known for their main cargo, the platelet and leukocyte receptors von‐Willebrand factor (VWF) and P‐selectin, WPB play a crucial role in hemostasis and inflammation. Here, the authors identify the glycerophosphodiester phosphodiesterase domain‐containing protein 5 (GDPD5) as a WPB cargo protein and show that GDPD5 is transported to WPB following uptake from the plasma membrane via an unique endocytic transport route. GDPD5 cleaves GPI‐anchored, plasma membrane‐resident proteins within their GPI‐motif, thereby regulating their local activity. The authors identify a novel target of GDPD5 , the complement regulator CD59, and show that it is released from the endothelial surface by GDPD5 following WPB exocytosis. This results in increased deposition of complement components and can enhance local inflammatory and thrombogenic responses. Thus, stimulus‐induced WPB exocytosis can modify the endothelial cell surface by GDPD5‐mediated selective release of a subset of GPI‐anchored proteins.

## Graphical abstract

Weibel‐Palade bodies (WPB) are unique secretory organelles of endothelial cells. A new WPB constituent is identified, the glycerophosphodiester phosphodiesterase domain‐containing protein 5 (GDPD5) and it is shown that GDPD5 is released to the cell surface upon stimulation of WPB exocytosis. Following release GDPD5 cleaves a subset of cell surface GPI‐anchored proteins and CD59 is identified as one of the GDPD5 targets .

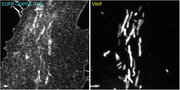

## 1 Introduction

Vascular endothelial cells rapidly respond to insults such as blood vessel injury or infection by releasing a variety of factors that regulate blood clotting and local immune responses. These factors include the platelet and leukocyte receptors von‐Willebrand factor (VWF) and P‐selectin that are stored in Weibel‐Palade bodies (WPB), rod‐shaped secretory granules unique to endothelial cells that can undergo evoked exocytosis following endothelial stimulation.^[^ ^1^, ^2^, ^3^ ^]^ WPB are lysosome‐related organelles that emerge at the trans‐Golgi network in a process driven by their main cargo, VWF. Further maturation of WPB then involves acidification, multimerization of VWF and acquisition of additional cargo proteins such as the P‐selectin cofactor CD63, which is delivered to WPB from late endosomal/lysosomal compartments. WPB maturation is believed to occur during microtubule‐dependent transport of the organelles to the cell periphery, where the mature WPB are anchored at the peripheral actin cytoskeleton (for reviews see^[^ ^4^, ^5^, ^6^, ^7^ ^]^). Stimulation by agonists like histamine, thrombin or epinephrine then triggers exocytosis of these WPB and thereby the release of their leukocyte and platelet adhesion promoting contents.^[^ ^8^, ^9^ ^]^ The complex exocytic fusion of WPB with the plasma membrane requires the participation of multiple proteins including Rab GTPases (i.e., Rab27A, Rab3B/D, Rab35), SNAREs and their interacting proteins (i.e., VAMP3/8, STXBP 1/5, MUNC13‐2/4) as well as other factors (ANXA2, S100A10, PLD1) that likely act in concert.^[^ ^10^, ^11^, ^12^, ^13^, ^14^, ^15^, ^16^, ^17^, ^18^ ^]^

The complexity of WPB as an organelle has been revealed by proteomic approaches, using density gradient centrifugation to isolate VWF‐containing WPB, or employing APEX2‐based proximity labelling to biotinylate and isolate WPB‐associated proteins.^[^ ^14^, ^19^, ^20^ ^]^ Glycerophosphodiester phosphodiesterase domain‐containing protein 5 (GDPD5) was reported to be significantly enriched in WPB in two approaches although a direct association with WPB had not been addressed.^[^ ^14^, ^19^ ^]^ GDPD5, also known as glycerophosphodiester phosphodiesterase 2 (GDE2), is a GDE family member acting in a phospholipase C‐like manner (for review see^[^ ^21^ ^]^). It consists of six transmembrane helices, a large catalytic ectodomain and a cytoplasmic terminal tail.^[^ ^22^ ^]^ In undifferentiated neuronal cells, it has been shown that after synthesis at the ER, GDPD5 is transported to the plasma membrane and later endocytosed and recycled via Rab4 and Rab11 positive endosomes. Most likely, GDPD5 functions exclusively at the plasma membrane where it has been reported to participate in choline metabolism and catalyze the cleavage of glycosylphosphatidylinositol‐anchored proteins (GPI‐APs). Three potential substrates of GDPD5 have been identified so far, the GPI‐anchored proteins reversion‐inducing cysteine‐rich protein with Kazal motifs (RECK), glypican 6 (GPC6) and GPC3, and cleavage of RECK by GDPD5 has been shown to induce motor neuron differentiation and neurogenesis by inactivation of Notch signaling in neighboring cells.^[^ ^23^, ^24^ ^]^ Moreover, a GDPD5‐mediated shedding of RECK has been reported to control the ADAM10/α‐secretase‐mediated cleavage of amyloid precursor protein (APP).^[^ ^25^ ^]^ However, other GDPD5 substrates have not been identified so far and a role of the enzyme in other cell types and tissue has not been addressed. Furthermore, a potential regulation of GDPD5 expression and activity on the cell surface has not been examined.

Here, we show that GDPD5 is a WPB resident protein in human endothelial cells, which is recruited to the secretory organelle via an unique endosomal transport route. We also identify a so far unknown GPDP5 substrate, the complement system regulator CD59, and show that CD59 is specifically released from the endothelial cell surface upon stimulation of WPB exocytosis and the resulting presentation of GDPD5 on the cell surface. Thus, GDPD5 is a novel WPB cargo that can modify the endothelial cell surface following evoked WPB exocytosis by cleaving a subset of GPI anchored proteins in a stimulus dependent manner.

## 2 Results

### 2.1 GDPD5 Localizes to WPB in Endothelial Cells

**Figure 1:**
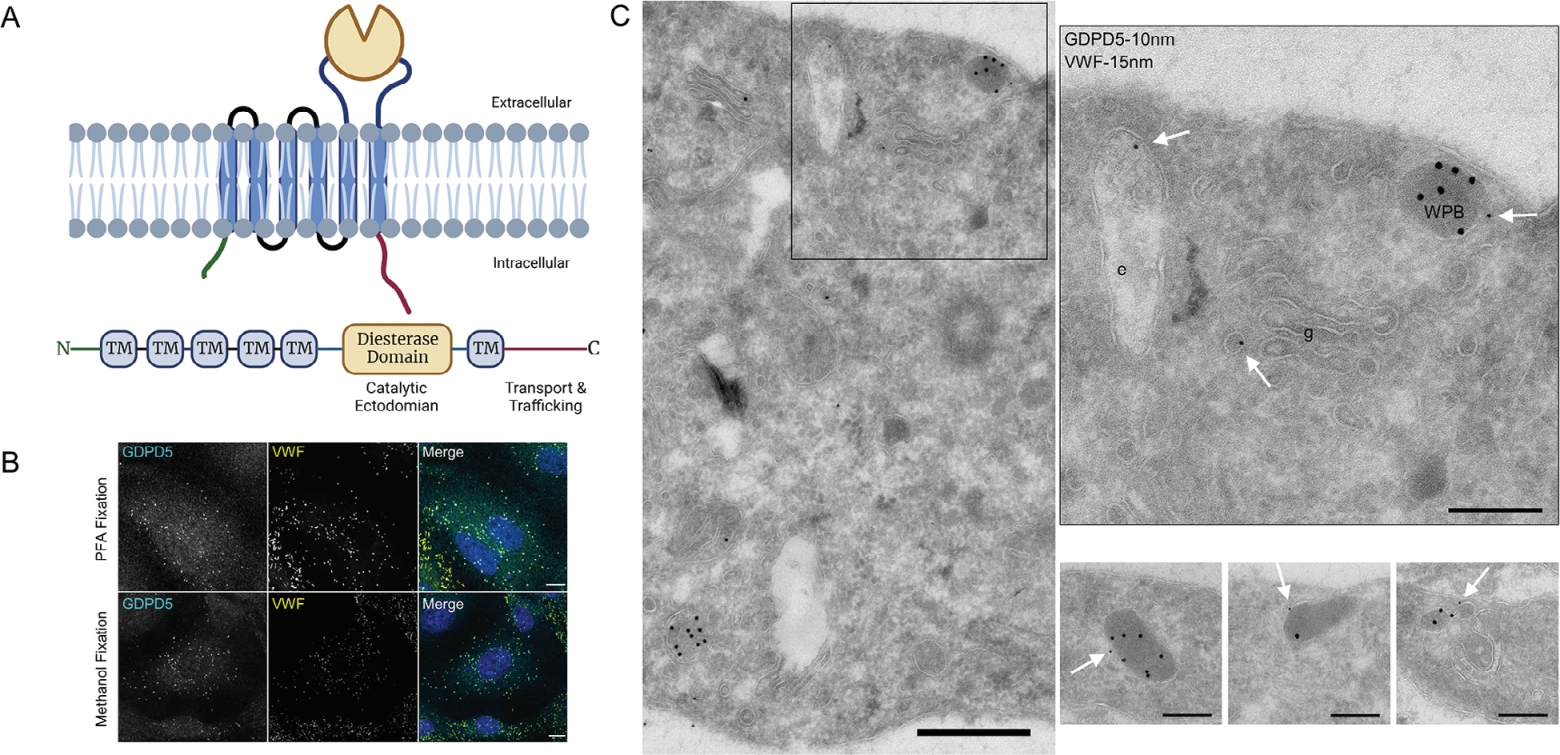
Localization of endogenous GDPD5 in HUVEC. A) GDPD5 embedded in the plasma membrane. GDPD5 consists of six transmembrane helices, a large catalytic ectodomain and cytoplasmic terminal tail (A, upper part). Domain structure of GDPD5 with transmembrane (TM) domains, catalytic diesterase domain and intracellular terminal tails (A, lower part). These figures were generated using BioRender. B) Colocalization of endothelial GDPD5 and VWF. HUVEC were cultivated for 24 h and fixed with 4% PFA or methanol (detailed protocol in methods section). Following permeabilization, cells were stained using the respective antibodies directed against VWF or GDPD5. Scale bar: 10 µm. C) Ultrastructural localization of endogenous GDPD5 in HUVEC immuno‐gold labelled on ultrathin cryosections with 10 nm protein A gold (processed according to the Tokuyasu‐method). The left panel shows a representative overview picture of subcellular structures. The black box is shown at higher magnification in the upper right image. Labelling for GDPD5 (10 nm protein A gold, white arrows) is found in endosomes (e), small transport vesicles, here close to the Golgi (g) and in colocalization with WPB, labelled for VWF with 15 nm protein gold. Lower right panels show more examples of colocalizations between endogenous GDPD5 and VWF clearly recognizable above a minimal background. As expected, the GDPD5 label is exclusively found at the rim of the membrane surrounding WPB, whereas the VWF label resides inside the WPB. Scale bar (left panel): 500 nm, Scale bar (right panels): 200 nm.

First, we assessed whether the phosphodiesterase GDPD5 (domain structure depicted in **Figure** **1**A) is a hitherto unknown cargo of WPB. In line with the proteomic data, immunofluorescence analysis revealed the presence of endogenous GDPD5 on VWF‐positive WPB of primary human endothelial cells (HUVEC) (Figure 1B). This was corroborated for the endogenous protein at the ultrastructural level. Electron microscopy of HUVEC stained for VWF and GDPD5 by immunogold labelling showed that GDPD5 is present at the limiting membrane of VWF‐positive structures (WPB) in addition to showing an association with the plasma membrane (PM) and endosomal structures (Figure 1C).

**Figure 2:**
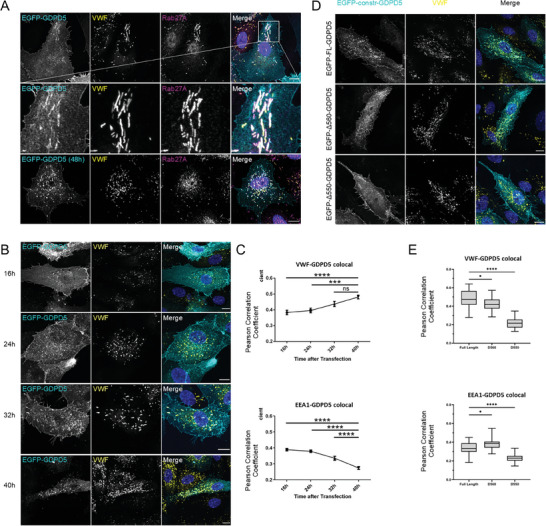
Localization and transport of EGFP‐GDPD5 in HUVEC. A) HUVEC were transfected with EGFP‐GDPD5 and fixed 24 h (or 48 h, where indicated) after transfection. Following permeabilization, cells were stained using the respective antibodies directed against VWF and Rab27A as WPB markers. Samples were analyzed by confocal laser scanning microscopy. Scale bar: 10 µm. B‐E) EGFP‐GDPD5 is found on WPB after endocytosis. HUVEC were transfected with EGFP‐GDPD5 and fixed at the indicated time points after transfection. Following permeabilization, cells were immunostained for VWF as a WPB marker and analyzed using confocal laser scanning microscopy. Scale bar: 10 µm (B). Colocalization between EGFP‐GDPD5 and the markers indicated was analyzed using Pearson correlation coefficient. Error bars = SEM, *n* ≥ 26 cells from 3 different experiments. Significance was tested using ordinary one‐way ANOVA with Dunnetts test for multiple comparisons (EEA1) or Kruskal Wallis with Dunn's test for multiple comparisons (VWF). (*** *p* < 0.001, **** *p* < 0.0001, ns = not significant) (C). HUVEC were transfected with the different EGFP‐GDPD5 constructs indicated (full length: FL, and the C‐terminal truncation mutants Δ560 and Δ550) and fixed 32 h post transfection. Cells were then permeabilized, immunostained for VWF as a WPB marker and EEA1 as an early endosome marker (no example shown for EEA1, colocalization only depicted in panel E) using the appropriate antibodies and analyzed using confocal laser scanning microscopy. Scale bar: 10 µm (D). Colocalization between the different EGFP‐tagged GDPD5 constructs, and the indicated marker was analyzed using Pearson correlation coefficient. *n* ≥ 31 cells from 3 different experiments. Significance was tested using ordinary one‐way ANOVA with Dunnetts test for multiple comparisons (VWF) or Kruskal Wallis with Dunn's test for multiple comparisons (EEA1). (* *p* < 0.05, **** *p* < 0.0001, ns = not significant) (E).

To record the dynamics of GDPD5 in live cells, we also expressed EGFP tagged versions of human GDPD5 in primary human endothelial cells (HUVEC) and first analyzed their localization 24 or 48 h after transfection. **Figure** **2**A shows that a substantial fraction of EGFP‐GDPD5 (EGFP fused to the N‐terminal end of GDPD5) localized to VWF‐positive WPB at these time points, in addition to some GDPD5 being present at the plasma membrane and in punctate structures identified as early endosomes by containing with antibodies directed against EEA1 (Figure S1, Supporting Information). Little to no colocalization with the late endosomal marker LAMP1 was observed (Figure S1, Supporting Information). Interestingly, at earlier time points post transfection, GDPD5 mainly localized to the ER (4 h), then the trans‐Golgi network (TGN; 8 h) and later to the plasma membrane and early endosomes (16 h) (Figure S1, Supporting Information). Identical localizations were observed for a GDPD5‐EGFP construct, in which the EGFP tag was fused to the C‐terminal end of GDPD5 (Figure S1, Supporting Information).

The localization data suggested that GDPD5 is not directly sorted to immature WPB at the TGN, but instead is first transported to the plasma membrane and then recruited to WPB following endocytosis. To verify this hypothesis, we performed additional time course and colocalization experiments. HUVEC were again transfected with EGFP‐GDPD5, fixed 16, 24, 32, or 40 h after transfection and subsequently stained for VWF as WPB marker or EEA1 as early endosomal marker. At the earliest time point analyzed in this series of experiments (16 h), a strong plasma membrane and early endosome localization was apparent (Figure 2B; Figure S1, Supporting Information). At later time points (24, 32 h), GDPD5 localized more to VWF‐positive WPB and some punctate structures in the cytoplasm. 40 h after transfection, GDPD5 mainly localized to WPB with only little plasma membrane staining visible (Figure 2B). No colocalization with the late endosome/lysosome (LE/L) marker LAMP1 was observed at any time (Figure S1, Supporting Information). The degree of colocalization between GDPD5 and the respective markers was also calculated employing the Pearson correlation (Figure 2C) and Manders overlap coefficients (Figure S1, Supporting Information) corroborating that the colocalization of GDPD5 and VWF‐positive WPB increased over time, whereas the colocalization between GDPD5 and early endosomes decreased. It should be noted here that absolute correlation/overlap coefficients are likely reduced by some general cellular background signal of the GDPD5 construct.

### 2.2 Transport of GDPD5 to WPB occurs after Endocytosis

So far, WPB constituents have been shown to be loaded onto the maturing organelle at the level of the Golgi/TGN (VWF) or via transport from late endosomes/lysosomes (LE/L, e.g. CD63^[^ ^7^ ^]^). As our data suggested a trans‐endosomal delivery route of GDPD5 to WPB not involving LE/L, we next analyzed the structural determinants of this transport route by generating C‐terminal truncation mutants of GDPD5 that had been shown to affect its trafficking in neuronal cells.^[^ ^22^ ^]^ Full length GDPD5 consists of 605aa, with an 88aa C‐terminal cytosolic tail, which is important for proper delivery to and from the cell surface (Figure 1A). Truncations in this C‐terminal tail were achieved by introducing stop codons after amino acids 560 or 550, respectively. In neuroblastoma cells, the Δ560 mutant has been reported to use a different recycling pathway and the Δ550 mutant loses its ability to be endocytosed. As revealed by immunostaining of HUVEC expressing the different GDPD5 constructs 32 h post transfection, the Δ560 mutant resembled full length GDPD5 in its distribution, whereas the Δ550 mutant showed an almost exclusive plasma membrane localization with only few intracellular punctate structures and no costaining with VWF indicating that endocytosis from the plasma membrane is required for WPB delivery (Figure 2D; see also Pearson correlation, Figure 2E, and Manders overlap coefficient, Figure S1, analyses, Supporting Information). At this time point post transfection (32 h), some limited colocalization with the endosomal marker EEA1 was observed for full length and Δ560 GDPD5, whereas colocalization was basically absent in case of the Δ550 mutant, indicating that this mutant indeed was not endocytosed. Note again that the Pearson correlation coefficients likely underestimate the degree of WPB colocalization due to some cellular background signal of the GDPD5 constructs, because no microscopic evidence for a visible colocalization of the Δ550 mutant with VWF‐ or EEA1‐positive structures could be obtained.

**Figure 3:**
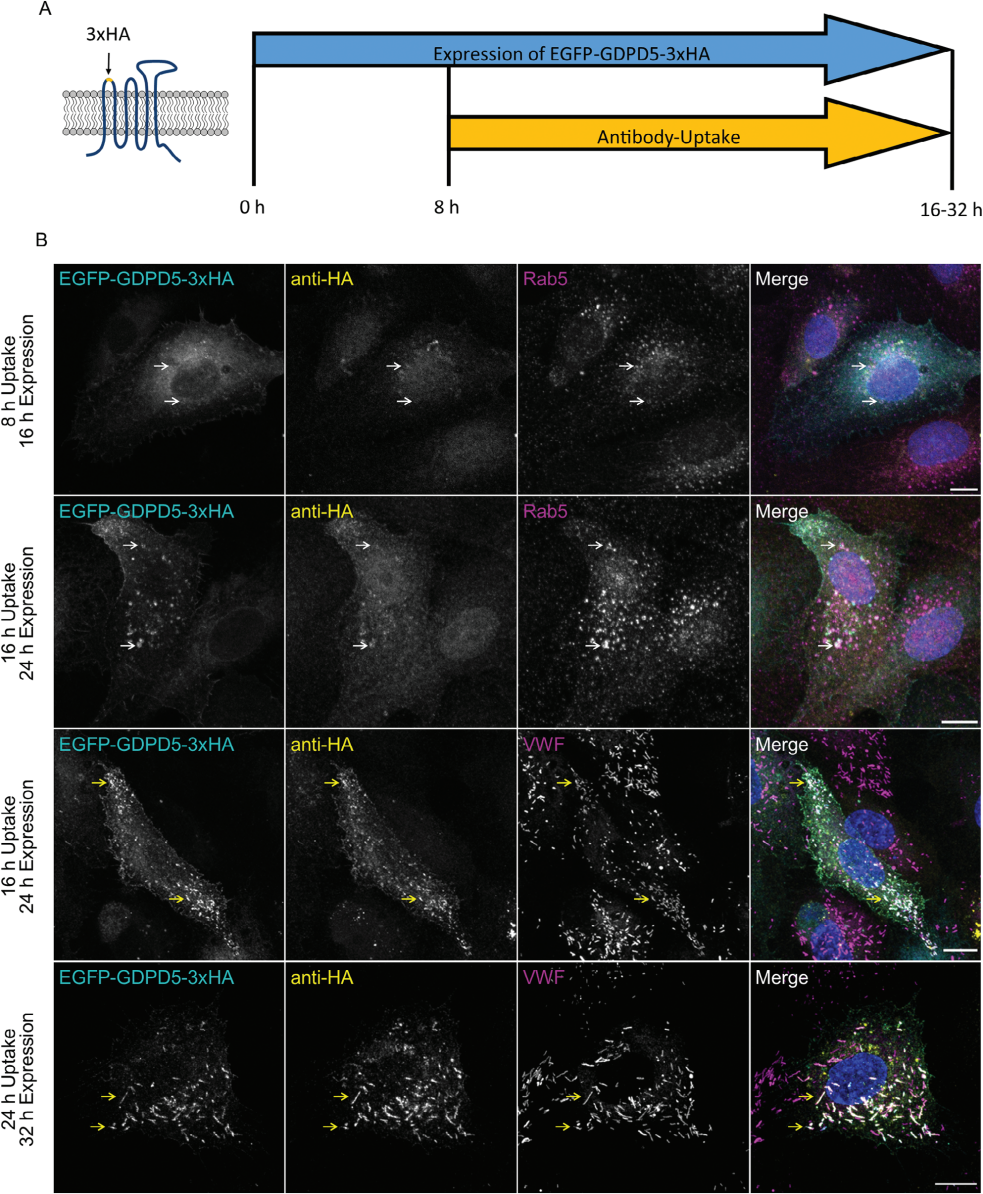
Antibody uptake assay revealing the transport route of endocytosed GDPD5. A) Schematic overview of the anti‐HA tag antibody uptake assay. GDPD5‐3xHA‐EGFP carrying a triple HA tag in the first extracellular loop was ectopically expressed in HUVEC. 8 h post transfection, a monoclonal anti‐HA tag antibody was added to the medium, and the cells were kept for another 8–24 h, washed and then fixed and permeabilized for subsequent immunofluorescence staining. B) Representative images of the anti‐HA tag antibody uptake in comparison to localization of the EGFP‐tagged GDPD5 construct and Rab5 as early endosome and VWF as WPB marker. White arrows mark Rab5 positive GDPD5 and HA‐antibody structures, yellow arrows mark VWF positive GDPD5 and HA‐antibody structures. Scale bar: 10 µm.

To confirm that the delivery of GDPD5 to WPB requires prior endocytosis from the plasma membrane, we established an antibody uptake assay. Therefore, a triple HA tag (3xHA) was included in the first extracellular loop of GDPD5. 8 h post transfection of HUVEC with EGFP‐3xHA‐GDPD5, an anti‐HA‐antibody was added to the medium and after an additional 8–24 h, cells were fixed and stained for VWF as WPB, Rab5 as early endosome and TGN46 as trans‐Golgi network marker (**Figure** **3**; Figure S2, Supporting Information). In this assay, anti‐HA antibodies are only found within intracellular vesicular structures if they had reacted with plasma membrane (PM)‐resident EGFP‐3xHA‐GDPD5 prior to its endocytosis and a transport from endosomes to WPB. Figure 3 shows that at early time points (8 and 16 h of antibody uptake) the anti‐HA antibody signal was mainly associated with the PM and early endosomes without significant TGN staining (Figure S2, Supporting Information). At 16 h uptake, some WPB were positive for the anti‐HA antibody, but the majority of the organelles remained negative suggesting that this is the earliest time at which antibody‐labelled EGFP‐3xHA‐GDPD5 can be detected in WPB (Figure 3B). 24 h after uptake, the majority of the EGFP‐3xHA‐GDPD5 construct and the anti‐HA antibody localized to WPB, indicating that the transport of GDPD5 to WPB indeed occurs after endocytosis of GDPD5 (Figure 3B). In control experiments, no anti‐HA antibody uptake is seen in cells expressing EGFP‐GDPD5, i.e., a construct lacking the HA tag (Figure S2, Supporting Information). To further characterize this unique transport route of GDPD5 in endothelial cells, we applied different inhibitors known to affect post‐endosomal trafficking in the antibody uptake assay. U18886A was chosen as a well‐characterized inhibitor of the late endosomal cholesterol transporter NPC1^[^ ^26^ ^]^ that had been shown before to interfere with trafficking routes emanating from late endosomes, TransNed19 as a NAADP receptor antagonist that inhibits NAADP mediated Ca^2+^‐release and endosomal transport^[^ ^27^ ^]^ and endosidin‐2 as a potent inhibitor of the exocyst complex subunit EXO70.^[^ ^28^ ^]^ Treatment with the different inhibitors had little if any effect on the trafficking of GDPD5; the internalized anti‐HA antibody still localized mostly to WPB, with some additional perinuclear structures only seen in the case of U18886A treatment (Figure S3, Supporting Information). These perinuclear structures were however not observed in the GFP channel, indicating an accumulation of the antibody alone.

**Figure 4:**
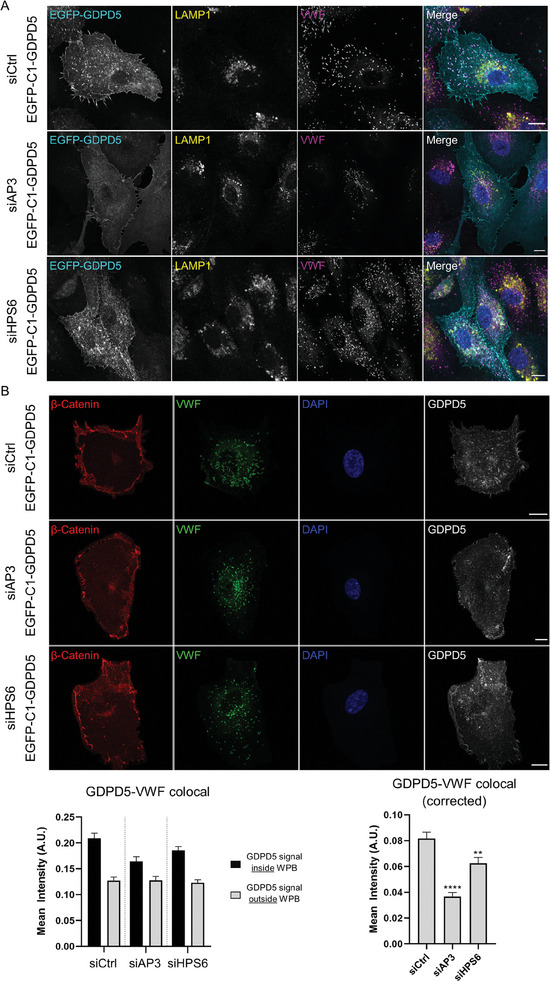
AP3 and HPS6 knockdown affect the localization of GDPD5. HUVEC were transfected with 400 pmol of the respective siRNA (siControl, siAP3 or siHPS6), kept for 48 h, transfected again with 400 pmol siRNA and EGFP‐C1‐GDPD5 respectively, and kept for 32 h. A) Cells were then subjected to immunofluorescence and stained for LAMP1 as late endosomal marker and VWF as WPB marker. Scale bar: 10 µm. B) Cells were then subjected to immunofluorescence and stained for β‐Catenin as cell border/contact marker and VWF as WPB marker. Bottom graphs) Colocalization between EGFP‐C1‐GDPD5 and VWF was analyzed using CellProfiler and the published Organelle Content Pipeline.^[^ ^34^ ^]^ EGFP‐GDPD5 signal intensities inside and outside VWF (WPB) are shown on the left (bar graphs with error bars = SEM) Corrected values (GDPD5 signal intensity inside WPB minus GDPD5 signal outside WPB) are shown on the right (bar graphs with error bars = SEM), *n* = 32 cells from 4 different experiments. Significance was tested using ordinary one‐way ANOVA with Dunnetts test for multiple comparisons. (** *p* < 0.01, **** *p* < 0.0001).

As the previous experiments suggested an unique trafficking route for the loading of GDPD5 onto WPB (PM‐endosomes‐WPB), we further analyzed this route by addressing the potential involvement of certain endosome and WPB associated proteins. Specifically, we focused on known (endosomal) transport proteins that were either significantly enriched in the WPB associated proteome^[^ ^14^ ^]^ – VPS51, Rab11B, MON2 or EHD1 – or known to mediate endosomal transport – VIPAS39, PI4Ks (PI4KIIα/β). Moreover, we analyzed factors already reported to participate in late endosome to WPB transport of CD63 and the vATPase subunit ATP6V0D1 – HPS6 and AP3β1, and we targeted the WPB associated Rabs GTPase Rab27A and Rab3B.^[^ ^29^, ^30^, ^31^, ^32^, ^33^ ^]^ Following siRNA mediated knockdown of the respective proteins (Figure S4, Supporting Information), colocalization of GDPD5 with VWF (WPB) or EEA1 (early endosomes) was analyzed microscopically and quantified using the Pearson correlation coefficient. Some reduction of the colocalization of GDPD5 and VWF was observed following knockdown of HPS6 and AP3 (**Figure** **4**A; Figure S4, Supporting Information) and to some extent also upon Rab27A depletion (Figure S4, Supporting Information, more evident in the microscopic images than in the Pearson correlation coefficient analysis that underestimates colocalization due to general cell and PM staining, see above). HPS6 depleted HUVEC also showed large, perinuclear accumulations of GDPD5, partly colocalizing with the late endosome/lysosome marker LAMP1 (Figure 4A; Figure S5, Supporting Information), whereas AP3 depleted HUVEC showed an overall reduction of intracellular GDPD5 positive vesicles (Figure 4A; Figure S5, Supporting Information). In addition to the Pearson analysis, colocalization of GDPD5 and VWF was quantified using CellProfiler with OrganelleContent Pipeline,^[^ ^34^ ^]^ confirming the reduced colocalization of GDPD5 with WPB (VWF) upon AP3 and HPS6 knockdown (Figure 4B). No significant effect was observed following depletion of the WPB‐associated Rab3B and Rab11B although we cannot exclude that other Rab isoforms such as Rab11A compensate for the loss. The transient localization of GDPD5 to early endosomes was slightly reduced by depletion of PI4 kinases PI4KIIα/β, which however did not affect the transport of GDPD5 to WPB in a significant manner (Figure S4, Supporting Information). Together, these data suggest that GDPD5 is transported to maturing WPB in a process possibly involving HPS6 and AP3. GDPD5 is mistargeted to late endosomes/lysosomes in case WPB delivery is blocked (HPS6 knockdown) and seems to remain at the PM following AP3 depletion. It remains to be determined whether the observed deficiencies in WPB recruitment occur due to direct transport related processes or impaired WPB maturation, which is known to occur following HPS6 and Rab27A knockdown and could result in WPB incapable of accepting GDPD5.^[^ ^8^, ^29^, ^30^ ^]^

### 2.3 CD59 and TFPI are GPI‐Anchored Targets of GDPD5 in Endothelial Cells

**Figure 5:**
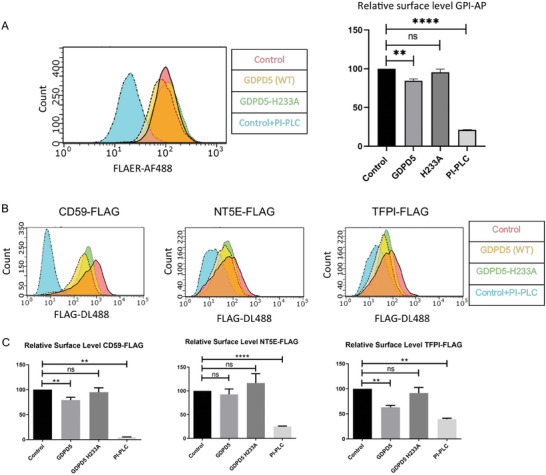
GDPD5 expression affects cell surface levels of certain GPI‐AP in endothelial cells. A) HUVEC were transfected with either mCerulean‐C1 empty vector (control), mCerulean‐GDPD5 or mCerulean‐GDPD5‐H233A, respectively, and subjected to a flow cytometry assay 24 h post transfection. Therefore, cells were detached, fixed and stained for the surface GPI‐AP signal using FLAER‐AF488. For each sample, 10000 mCerulean expressing cells were counted and analyzed for their AF488 fluorescence intensity. Histograms show FLAER‐AF488 intensity distributions with the right shifted curve indicating increased surface GPI‐AP (left). Quantification of the relative surface levels of GPI‐AP analyzed via flow cytometry of the different transfected cell populations is shown on the right (quantification based on median fluorescence intensity values detected above control antibody threshold). Error bars = SEM, *n* = 8 experiments. Significance was tested using repeated measures ANOVA with Dunnetts test for multiple comparisons (** *p* <0.01, **** *p* <0.0001, ns = not significant). B) HUVEC were co‐transfected with either mCerulean‐C1 empty vector (control), mCerulean‐GDPD5 or mCerulean‐GDPD5‐H233A and the respective FLAG‐tagged GPI‐AP target (CD59‐FLAG, NT5E‐FLAG or TFPI‐FLAG). 24 h post transfection, cells were detached, fixed and stained for the surface FLAG signal using anti‐FLAG(M1)‐DL488 antibodies. For each sample, 10000 mCerulean expressing cells were counted and analyzed for their DL488 fluorescence intensity. Histograms show the FLAG‐DL488 intensity distribution with a right shift indicating more surface localized GPI‐AP. C) Quantification of the data shown in A. Error bars = SEM, *n* ≥ 7 experiments. Significance was tested using ordinary one‐way ANOVA with Dunnetts test for multiple comparisons. Paired Wilcoxon test or paired t‐test were used when the number of experiments wasn't the same for all samples. (** *p* <0.01, **** *p* <0.0001, ns = not significant).

GDPD5 is a phosphodiesterase that can cleave GPI‐anchors of certain proteins and thereby plays an important role in regulating their cell surface activity. To date, 3 targets of GDPD5 are known, RECK, GPC3 and GPC6, however, no GDPD5 mediated target cleavage has been analyzed in endothelial cells.^[^ ^23^, ^24^ ^]^ To address the enzymatic role of GDPD5 in endothelial cells, we first verified that the enzyme is also involved in the cleavage of GPI‐AP in these cells. Therefore, HUVEC were transfected with mCerulean control vector, mCerulean‐GDPD5 or the mutant protein mCerulean‐H233A‐GDPD5 which has suffered a H233A substitution rendering the protein enzymatically less active.^[^ ^24^ ^]^ This ectopic expression approach employing the H233A‐GDPD5 mutant was chosen to unequivocally relate any GPI‐AP cleavage observed to GDPD5. As a positive control, cells were treated with PI‐PLC, which effectively cleaves all GPI anchors. Cells were then subjected to FACS analysis for their mCerulean expression level and their surface GPI‐AP level employing fluorescently labelled aerolysin (FLAER‐AF488), which binds to GPI anchors.^[^ ^35^, ^36^ ^]^ A significant reduction of GPI‐AP surface levels was observed in GDPD5 overexpressing cells compared to the control conditions or the less active point mutant, respectively. However, the reduction was significantly lower than in PI‐PLC treated cells suggesting that GDPD5 only cleaves a subset of GPI anchors (**Figure** **5**A).

**Figure 6:**
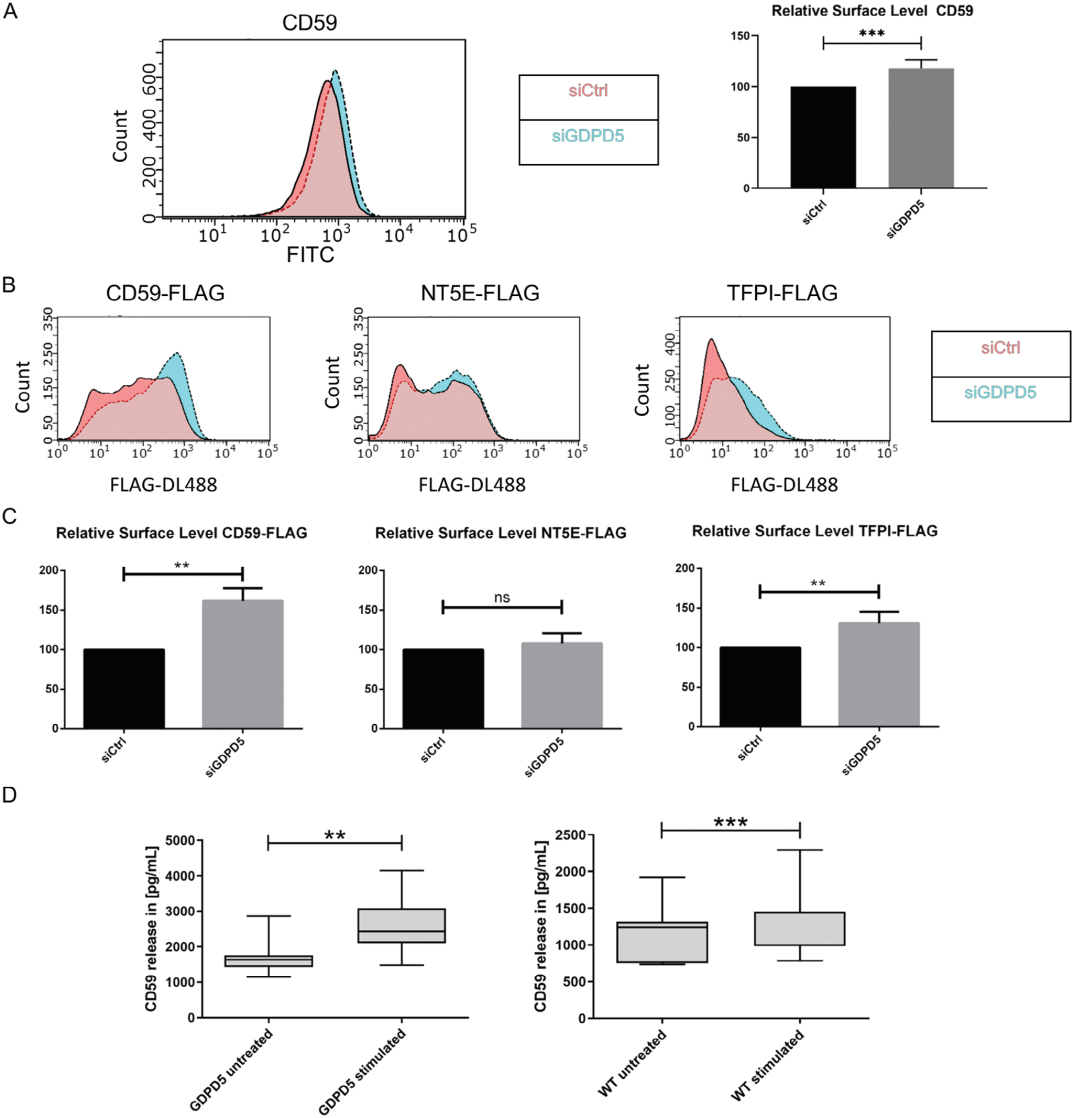
CD59 and TFPI are targets of GDPD5 on the endothelial cell surface. A) HUVEC were transfected with 200 pmol of siCtrl or siGDPD5, kept for 48 h, and transfected again with the same amounts of the respective siRNAs. 24 h after the second transfection, HUVEC were detached, fixed and stained for the surface CD59 level using anti‐CD59‐FITC antibodies. For each sample, 20000 cells were counted and analyzed for their FITC fluorescence intensity. B) HUVEC were transfected with 200 pmol of siCtrl or siGDPD5, kept for 48 h, and transfected again with the same amounts of the respective siRNAs and the GPI‐AP target indicated. 24 h after the second transfection, HUVEC were detached, fixed and stained for the surface FLAG signal using anti‐FLAG(M1)‐DL488 antibodies. For each sample, 20000 cells were counted and analyzed for their DL488 fluorescence intensity. C) Quantification of the data shown in B (carried out as described in legend to Figure 5). Error bars = SEM; *n* = 5 for NT5E‐FLAG; n ≥ 8 experiments for CD59‐FLAG or TFPI‐FLAG. Significance tested with paired t‐test or paired Wilcoxon test (** *p* < 0.01, ns = not significant). D) HUVEC were either transfected with GDPD5‐EGFP or left untransfected and then cultured for 32 h. Cells were washed and subsequently incubated with starvation medium for 1 h. Medium was collected (untreated samples) and cells were incubated with starvation medium supplemented with 500 µM histamine for 1 h. Medium was again collected (stimulated samples) and samples were analyzed for their soluble CD59 content via CD59‐ELISA. *n* = 10, significance tested with paired Wilcoxon test (left) or paired t‐test (middle, right) (** *p* < 0.01, *** *p* < 0.001).

To identify specific targets of GDPD5 in HUVEC, we first determined by RT‐PCR which GPI‐anchored proteins are expressed in endothelial cells and among them chose three candidates with known functions in endothelial cell biology for further analysis, CD59, NT5E (CD73) and TFPI (Figure S6, Supporting Information). CD59, also known as membrane attack complex (MAC)‐inhibitory protein, is an inhibitor of the complement reaction and protects cells from deposition of the terminal complement complex (TCC or MAC) which can initiate a pro‐inflammatory activation of the endothelium.^[^ ^37^, ^38^ ^]^ NT5E is a 5′ nucleotidase and can hydrolyse AMP to yield the anti‐inflammatory adenosine.^[^ ^39^ ^]^ Tissue factor pathway inhibitor (TFPI) is involved in the regulation of blood coagulation by binding to and inhibiting factor Xa and subsequently also the factor VIIa‐TF complex.^[^ ^40^ ^]^ To study the potential involvement of GDPD5 in the GPI anchor cleavage of these target candidates, we first expressed versions of CD59, NT5E and TFPI harbouring a FLAG tag in the extracellular protein domain together with one of the above mentioned mCerulean constructs, i.e., mCerulean (as control), mCerulean‐GDPD5 or mCerulean‐H233A‐GDPD5. FACS analysis of the respective HUVEC 24 h post transfection, which employed a FLAG‐DL488 antibody for determining surface levels of the FLAG‐tagged targets, revealed that CD59 and TFPI but not NT5E experienced a significant reduction of their surface levels in cells overexpressing wild‐type GDPD5, but not in H233A‐GDPD5 mutant expressing cells (Figure 5B,C). To further confirm this, we performed a similar flow cytometry assay for cell surface protein levels with HUVEC depleted of GDPD5 by siRNA‐mediated knockdown (Figure S6, Supporting Information). **Figure** **6**A–C show that this treatment resulted in a significant increase in the surface levels of endogenous CD59, as well as FLAG‐tagged CD59 and TFPI but not NT5E, supporting the notion that CD59 and TFPI are specific targets of GDPD5.

**Figure 7:**
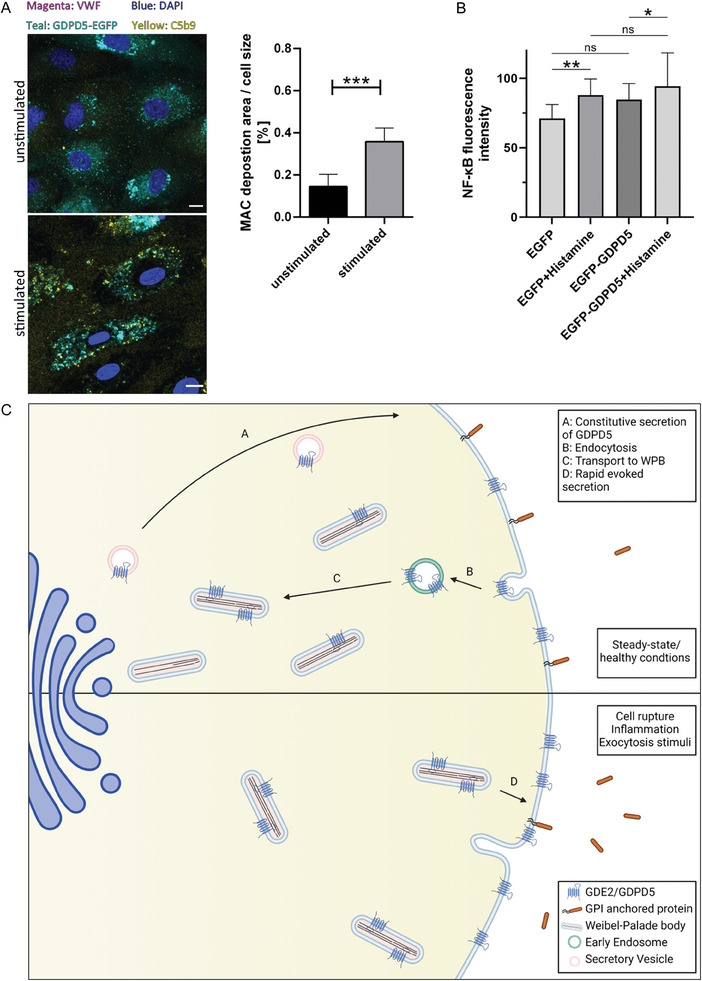
GDPD5 affects MAC deposition on HUVEC surfaces and pro‐inflammatory nuclear translocation of NFκB. A) HUVEC were transfected with GDPD5‐EGFP and cultured for 32 h. Cells were then treated with 20% human serum for 4 h and either left unstimulated or stimulated for 1 h with histamine (500 µM). Subsequently, cells were fixed and stained for C5b‐9 (yellow) as part of MAC and VWF (magenta, only upper panel) as WPB marker. GDPD5‐EGFP is shown in teal and nucleus/DAPI is shown in blue (A, left). MAC deposition area was calculated using Fiji software. n = 32 cells from 4 independent experiments. Significance was tested using Mann Whitney test (*** *p* < 0.001) (A, right). B) HUVEC expressing EGFP or EGFP‐GDPD5 were treated with 30% human serum with or without histamine (500 µM) for 2 h and subsequently subjected to immunostaining with antibodies directed against the p65 subunit of NFκB (see Figure S9, Supporting Information for examples of the immunostaining). Nuclear anti‐p65 antibody fluorescence intensity (nuclei identified by DAPI staining) was determined in *n* = 43 (EGFP), *n* = 55 (EGFP+Histamine), *n* = 42 (EGFP‐GDPD5) and *n* = 49 (EGFP‐GDPD5+Histamine) cells. Bars indicate median and error bars show 95% confidence interval. Statistics employed Kruskal‐Wallis and Dunn's multiple comparison tests (** *p* < 0.01, * *p* < 0.05, ns = not significant). C) Model depicting GDPD5 transport and function in endothelial cells. GDPD5 is continuously secreted via biosynthetic transport (A) and endocytosed into early endosomes (B). Internalized GDPD5 is then transported from early endosomes to WPB (C) where it is stored for a subsequent regulated release upon stimulation (D). At steady–state most GDPD5 is localized to WPB. Once this large fraction is acutely released to the cell surface via WPB exocytosis, it cleaves a specific subset of GPI anchors and thereby changes the activity of these GPI anchored proteins on the endothelial cell surface. Figure was generated using BioRender.

Due to its role as important complement regulator, we then focused on CD59 and confirmed that the endogenous protein is a GDPD5 substrate because the amount of soluble CD59 released into the culture medium is significantly increased in GDPD5 overexpressing as compared to WT HUVEC (Figure 6D). Next, we assessed whether endothelial stimulation which is known to trigger WPB exocytosis affects the cell surface levels of GPI‐anchored CD59. Therefore, we first verified that the secretion of GDPD5‐positive WPB can be evoked by stimulation with histamine or VEGF/ATP (Figure S7, Supporting Information). Next, we showed that histamine stimulation and thus WPB exocytosis triggered a release of endogenous CD59 from endothelial cells into the culture medium and that this release is enhanced in GDPD5 overexpressing compared to WT cells (Figure 6D). As these results identify CD59 as a specific GDPD5 target that is subject to GPI anchor cleavage in histamine stimulated HUVEC, we analyzed downstream effects of this elevated CD59 release. Therefore, we used an established assay to determine cell surface assembly of the membrane attack complex (MAC), which is known to be regulated by CD59.^[^ ^38^ ^]^ HUVEC were transfected with GDPD5‐EGFP and then treated with 20% human serum as source of MAC components with or without stimulating the cells with histamine. As revealed by staining for MAC deposition, histamine stimulation resulted in an increase of regions showing a strong MAC signal (**Figure** **7**A). However, even in these stimulation conditions the cells showed no significant increase in cell lysis (Figure S8, Supporting Information) suggesting that the increased MAC deposition, which is known to trigger a pro‐inflammatory activation of the endothelium (for review see^[^ ^41^ ^]^), is not sufficient to cause osmolysis. To assess a potential pro‐inflammatory activation following WPB exocytosis and the resulting surface delivery of GDPD5, CD59 release and MAC deposition, we used nuclear translocation of the p65 subunit of NFκB as a readout. HUVEC expressing EGFP (control) or EGFP‐GDPD5 subjected (or not) to histamine stimulation and treatment with human serum were analyzed by immunofluorescence employing anti‐p65 antibodies. Figure S9 (Supporting Information) (example) and Figure 7B (quantification) show that histamine stimulation increases the nuclear translocation of p65 and that this effect is slightly more pronounced in cells overexpressing EGFP‐GDPD5 although the latter increase is statistically not significant. Together these results indicate that histamine evoked release of GDPD5 to the cell surface, which occurs by WPB exocytosis, leads to lower cell surface levels of CD59 and in turn an increased (non‐lytic) MAC deposition, which likely acts pro‐inflammatory.

## 3 Discussion

In this study we identified GDPD5 as a novel WPB cargo that following cell surface delivery by WPB exocytosis can modify the surface of endothelial cells by modulating the repertoire of GPI‐AP. GDPD5 is transported to WPB via an endosomal transport route that involves internalization from the plasma membrane into early endosomes and subsequent delivery to WPB. Following secretagogue stimulation of WPB exocytosis, the GDPD5 stored in WPB is exported to the cell surface where it acts by cleaving a subset of GPI‐AP within their GPI‐motif, and we could identify two so far unknown targets of GDPD5, CD59 and TFPI, which are cleaved on the endothelial cell surface.

We analyzed the transport route of GDPD5 in HUVEC using trafficking mutants, a newly developed antibody uptake assay as well as depletion of endosomal transport proteins. Thereby, the transport route could be defined as follows: Following synthesis, GDPD5 is first transported to the PM (possibly a default pathway), then internalized into EEA1 positive early endosomes and finally transferred to WPB, where the enzyme is stored for subsequent regulated release. At steady state, rather low surface and endosome levels of GDPD5 are observed and the majority of the protein is localized to WPB. This permits an acute release to the cell surface in emergency situations like vascular injury or inflammation and thus a specific and regulated remodeling of cell surface GPI‐APs in these situations (Figure 7C).

Other known cargo proteins that are not directly copacked into WPB at the level of the Golgi are the tetraspanin CD63, which is transported via LE/L in a BLOC‐2/exocyst, AP3 and annexin A8 dependent manner, and the v‐ATPase subunit ATP6V0D1, which requires HPS6/BLOC‐2 for transport to WPB.^[^ ^29^, ^30^, ^33^, ^42^ ^]^ As GDPD5 shows almost no localization to LE/L and its delivery to WPB is not affected by perturbation of post‐LE/L trafficking with the NPC inhibitor U18886A, it likely uses a WPB delivery route that differs from the LE/L‐to‐WPB transport of CD63. Our results suggest that the GDPD5 transport to WPB rather occurs from early endosomes, as early endosomes positive for both, EEA1 and GDPD5 appear to reside in close proximity to WPB (Figure S10, Supporting Information) and the antibody uptake assay identifies early endosomes as an intermediate trafficking station of GDPD5 before it reaches the WPB. Interestingly, the protein machinery involved in this EE‐to‐WPB transport also seems to include constituents of the BLOC‐2 complex, as HPS6 knockdown impairs GDPD5 delivery to WPB and leads to a mistargeting of the protein to LE/L. In melanocytes, the BLOC‐2 complex is needed for cargo transport to their lysosome‐related organelles, the melanosomes, and here it operates by targeting recycling endosomal tubules to melanosomes.^[^ ^43^ ^]^ On the other hand, late endosomal transport tubules appear to exist in close proximity to WPB where they mediate the BLOC‐2 dependent transport of CD63 and the v‐ATPase subunit ATP6V0D1.^[^ ^29^, ^30^ ^]^ However, the EE‐resident GDPD5 that is found in close proximity to WPB does not appear to reside in tubular structures (Figure S10, Supporting Information). AP3 knockdown, which also has been shown to affect CD63 transport to WPB, also resulted in an impaired delivery of GDPD5 to WPB.^[^ ^33^ ^]^ However, the EE to WPB transport of GDPD5 appears to differ from the CD63 transport route, as GDPD5 seems to be trapped at the plasma membrane upon AP3 depletion whereas the same treatment resulted in an accumulation of CD63 in LE/L (Figure 4; Figure S5, Supporting Information).^[^ ^33^ ^]^

A function of GDPD5 in the endothelium has not been reported so far and we show for the first time that it can cleave a subset of cell surface GPI‐APs in these cells. Interestingly, as also described earlier for other cells,^[^ ^23^, ^24^ ^]^ the enzyme shows specificity for certain GPI‐APs as only a fraction of the endothelial GPI‐APs that can be released by PI‐PLC are cleaved by GDPD5. The endothelial cell surface targets identified herein are CD59 and TFPI, which are both participating in the regulation of complement, inflammatory and coagulation reactions. CD59 is an inhibitor of the MAC‐complex and is related to various diseases including paroxysmal nocturnal haemoglobinuria and obstructive sleep apnea.^[^ ^38^, ^44^, ^45^ ^]^ A recent study in HUVEC could show that in intermittent hypoxia conditions, CD59 plasma membrane presentation is impaired and consequently ECs are more accessible to complement attack and MAC deposition.^[^ ^46^ ^]^ Interestingly, MAC assembly on nucleated cells has been shown to be largely non‐cytolytic and on endothelial cells, it can trigger NLRP3 inflammasome formation and the release of pro‐inflammatory cytokines such as IL‐1β which promote leukocyte adhesion.^[^ ^38^, ^47^ ^]^ This pro‐inflammatory activity could be the result of only transient membrane pores formed by the MAC that permit K^+^ efflux and Ca^2+^ influx but are then efficiently removed by endocytosis and/or exocytotic membrane shedding.^[^ ^48^, ^49^ ^]^ The pore‐mediated Ca^2+^ influx would in turn lead to a sustained elevation of intracellular Ca^2+^ thereby further promoting WPB exocytosis when classical G protein‐coupled receptor pathways become desensitized after a few minutes of stimulation.^[^ ^50^ ^]^ Only when pores become overwhelming, apoptotic cell death will be initiated which could be beneficial to the vasculature by removing such overreacting cells from the endothelium. In this respect it is worth noting that a non‐lytic assembly of MAC promotes WPB exocytosis and the release of highly multimeric VWF, and this has been connected to endothelial protection from complement lysis.^[^ ^51^, ^52^, ^53^ ^]^ Possibly this could be explained in part by the WPB resident and VWF interacting complement factor H, that has been shown to protect CD59 deficient erythrocytes from MAC deposition.^[^ ^54^, ^55^ ^]^ Hence, evoked WPB exocytosis not only supports a pro‐inflammatory milieu through the release of P‐selectin/CD63 and VWF, but it also renders the endothelium more susceptible to MAC deposition and additional inflammatory activation through GDPD5 secretion and the resulting CD59 shedding. TFPIβ, the GPI‐anchored isoform of TFPI, is the other endothelial GDPD5 target identified here. As TFPI inhibits the clotting factor Xa and the factor VIIa‐tissue factor complex, its GDPD5 mediated release from the endothelium likely supports the VWF‐induced blood clotting and platelet plug formation at sites of vascular injury, where WPB exocytosis had occurred.^[^ ^56^, ^57^ ^]^ Thus, multimeric VWF and GDPD5, which are simultaneously released by evoked WPB exocytosis, act in concert to support rapid blood clotting.

To date, three other targets of GDPD5 have been identified in other cells, RECK and glypicans 3 and 6.^[^ ^23^, ^24^ ^]^ Although their potentially regulated surface anchorage in endothelial cells has not been investigated, it is noteworthy that Notch signaling which is affected by RECK fulfills an important role in blood vessel development.^[^ ^58^, ^59^, ^60^ ^]^ In fact, RECK knockdown in HUVEC leads to altered vascular tube formation and cell senescence, as well as enhanced metalloproteinase and decreased β1‐integrin activity.^[^ ^61^ ^]^ It is possible that GDPD5 is involved in these processes as an upstream regulator of RECK activity coupling endothelial activation and WPB exocytosis to blood vessel development. Moreover, the activity of the shedases ADAM10 and ADAM17 toward their numerous targets, including ICAM‐1, VCAM‐1, VE‐Cadherin, Notch or VEGFR2, which is partly regulated by RECK, might therefore also be controlled indirectly by surface presentation of the RECK GPI anchor‐cleaving GDPD5.^[^ ^62^, ^63^, ^64^, ^65^ ^]^ Thus, by selectively controlling the surface levels of specific GPI‐anchored proteins, WPB exocytosis and the resulting secretion of GDPD5 can regulate endothelial cell functions that go beyond VWF and P‐selectin externalization.

## 4 Experimental Section

### Cell Culture

HUVEC (Promocell, C‐12208) were cultured in a 1:1 (V:V) mixture of Endothelial Cell Growth Medium II (Promocell) and Medium 199 Earle's (Sigma) containing 10% fetal calf serum (Sigma), 30 µg mL^−1^ gentamycin (CytoGen) and 0.015 µg mL^−1^ amphotericin B (Biochrom) – Mix Medium – at 37 °C and 5% CO_2_ for up to 5 passages. Transfection of HUVEC with siRNA or plasmids was performed using the Amaxa Nucleofection Device (HUVEC Nuclefector Kit‐OLD, Lonza) as described previously.^[^ ^10^ ^]^

### Plasmids and siRNAs

siRNA‐mediated knockdown employed the following siRNAs: GDPD5^[^ ^66^ ^]^: 5′‐GCUCUCCGUAUGUUCAGACAA dTdT‐3′, Rab27A^[^ ^15^ ^]^: 5′‐GGAGAGGUUUCGUAGCUUA dTdT‐3′, MON2^[^ ^67^ ^]^: 5′‐CAU GCA GAU AAU GUA UCC AGC UA dTdT‐3, ′EHD1^[^ ^68^ ^]^: 5′‐GAA AGA GAU GCC CAA UGU C dTdT‐3′, VPS51^[^ ^69^ ^]^: 5′‐GCU AUU CUC UGA ACG UAU U dTdT‐3′, HPS6: Invitrogen Silencer® Select: 4 392 421 / s36366, Rab11B: siTools Biotech: 9230 – RAB11B (human), VIPAS39: siTools Biotech: 63 894 – VIPAS39 (human), PI4K2A^[^ ^70^ ^]^: 5′‐GGA UCA UUG CUG UCU UCA A dTdT‐3′, PI4K2B^[^ ^70^ ^]^: 5′‐GGU UCA AGU GGA AGU UAC U dTdT‐3′, AP3β1^[^ ^71^ ^]^: 5′‐AUG GCU GAU CUU GAA GGU UUA dTdT‐3′, Rab3B^[^ ^17^ ^]^: 5′‐GCA CAA CGU GCU UGU UUC C dTdT‐3′.

Plasmids were generated by PCR‐mediated amplification of the respective cDNA fragments from a self‐made HUVEC cDNA library and ligation of the appropriately digested fragments into commercially available vectors using the following primers:

Pr. Fw GDPD5 (SalI): 5′‐CGA **GTCGAC** GGCACGAGTATGGTGAGAC‐3′, Pr. Rev GDPD5 (BamHI): 5′‐TCA **GGATCC** CTTCAGCTAACGCCCACTC‐3′, Pr. Fw CD59 (XhoI): 5′‐GCT **CTCGAG** GTTCTGTGGACAATCACAATGG‐3′, Pr. Rev CD59 (EcoRI): 5′‐TCT **GAATTC** CCTGGTGTTGACTTAGGGATG‐3′, Pr. Fw NT5E (XhoI): 5′‐GTC **CTCGAG** GTTCACGCGCCACAGCTATG‐3′, Pr. Rev NT5E (EcoRI): 5′‐CTT **GAATTC** GCTGTCACAAAGCCAGGTCC‐3′, Pr. Fw TFPI (XhoI): 5′‐CAT **CTCGAG** CGCCAGTTTCTTGATCTGC‐3′, Pr. Rev TFPI (EcoRI): 5′‐ACA **GAATTC** AAGGAAATGCCAAAAGCAC‐3′.

Point mutations or insertions of protein tags were generated by the Q5 site‐directed mutagenesis kit (New England Biolabs) according to the manufacturer's instructions. The following primers were used:

Pr. Fw. GDPD5 Δ570: 5′‐TCTCCGTATGATCAGACAACAG‐3′, Pr. Rev. GDPD5 Δ570: 5′‐GCACATCGGAGACCTCTA‐3′, Pr. Fw. GDPD5 Δ560: 5′‐TGAGGTGTAGAGGTCTCCGAT‐3′, Pr. Rev. GDPD5 Δ560: 5′‐ATCGCTGATCTCTGAGAAAATAAG‐3′, Pr. Fw. GDPD5 Δ550: 5′‐TGAGAGAAGCTTATTTTCTCAGAG‐3′, Pr. Rev. GDPD5 Δ550: 5′‐CTTCATGATGCTGACGTC‐3′, Pr. Fw. GDPD5 Δ540: 5′‐TGAGACCAGCCGGGACGTCAG‐3′, Pr. Rev. GDPD5 Δ540: 5′‐GCGCACCGCAGCACTCAG‐3′, Pr. Fw. GDPD5 Δ530: 5′‐CTACAACCCTTAGCAGATCATGCTG‐3′, Pr. Rev. GDPD5 Δ530: 5′‐CTCCGTATGCCACCCAGG‐3′, Pr. Fw. GDPD5 H233A: 5′‐TCTCATTGGCGCCCGCGGGGCCCC‐3′, Pr. Rev. GDPD5 H233A: 5′‐GCAGGCTTGGGGCCGAGG‐3′, Pr. Fw CD59‐FLAG: 5′‐GATGATGATAAACTGCAGTGCTACAACTGTC‐3′, Pr. Rev CD59‐FLAG: 5′‐ATCTTTATAATCGCTATGACCTGAATGGCAG‐3′, Pr. Fw NT5E‐FLAG: 5′‐GATGATGATAAATGGGAGCTTACGATTTTGCACACCAACGACGTG‐3′, Pr. Rev NT5E‐FLAG: 5′‐ATCTTTATAATCGGCGCCAGCCGCAGGCCA‐3′, Pr. Fw TFPI‐FLAG: 5′‐GATGATGATAAAGATTCTGAGGAAGATGAAGAACAC‐3′, Pr. Rev TFPI‐FLAG: 5′‐ATCTTTATAATCAGCATTAAGAGGGGCAGG‐3′.

Insertion of 3xHA into the first extracellular loop of GDPD5 was achieved using the NEBuilder Hifi DNA Assembly according to manufacturer's instructions. The following primers were used:

Pr. Fw GDPD5: 5′‐GAATTCAACTGGTACCTC‐3′, Pr. Rev GDPD5: 5′‐ATCATAGTCATTGTGGAC‐3′, Pr. Fw 3xHA: 5′‐CACAATGACTATGATTATCCGTATGATGTGCCGGATTATGCGTATCC, GTATGATGTGCCGGATTATGCGTATCCGTATGATGTGCCGGACTATGCTGAATTC‐3′, Pr. Rev 3xHA: 5′‐GTACCAGTTGAATTCAGCATAGTCCGGCA‐3′

### Real‐Time PCR

RNA isolation from HUVEC employed the RNEasy Mini Kit (Qiagen). 1 µg of RNA per sample was transcribed into cDNA using High‐Capacity cDNA Reverse‐Transcription Kit (Thermo Fisher Scientific). Real‐Time PCR was performed with Brilliant III Ultra‐Fast SYBR Green qPCR Mastermix (Agilent) on a Roche LightCycler 480 according to the manufacturer's instructions. Analysis of the data was performed using the $2^{-\Delta \Delta C_{T}}$‐method.^[^ ^72^ ^]^

### LDH Cytotoxicity Assay

LDH Cytotoxicity Detection Kit was obtained from Takara Bio (#MK401) and the assay was carried out according to manufacturer's instructions. Cells were treated with 0.25% Triton in assay medium as positive control.

### Western Blotting

Cell lysates were prepared by treating HUVEC with RIPA buffer (25 mM Tris (pH 7–8), 150 mM NaCl, 0,1% SDS, 0,5% Natriumdeoxycholat, 1% Triton X‐100 + protease inhibitor) for 30 min on ice. Thereafter, cells were sonicated for 1 min at 4 °C for final lysis. After centrifugation at 10 000*g for 10 min, the supernatant was mixed with sample buffer and boiled for 10 min at 95 °C. Samples were then subjected to SDS‐PAGE and Western blotting according to standard protocols. Knockdown of overexpressed EGFP‐GDPD5 was confirmed by using the lysate of confluent 6 cm dishes 24 h after the second transfection with the respective siRNAs and EGFP‐GDPD5 plasmids.

Antibodies employed were as follows:

**Table:** 

Antibody	Manufacturer	Dilution (Application)
ms‐α‐VWF	DAKO	1:500 (IF)
ms‐α‐EEA1	BD Biosciences	1:800 (IF)
ms‐α‐LAMP1 (H4A3)	DSHB	1:200 (IF)
ms‐α‐CD63 (H5C6)	DSHB	1:200 (IF)
ms‐α‐β‐Catenin	BD Biosciences	1:500 (IF)
ms‐α‐FLAG®(M1)‐DL488	Sigma / Thermo Fisher	1:400 (FC)
ms‐α‐CD59‐FITC	Biolegend	1:40 (FC)
ms‐α‐HA	Biolegend	1:100‐1:200 (IF)
ms‐α‐α‐Tubulin	Invitrogen	1:10.000 (WB)
rb‐α‐VWF	DAKO	1:25 000 (IF), 1:100 (EM)
rb‐α‐Rab27A	Proteintech	1:150 (IF), 1:500 (WB)
rb‐α‐KDEL	ThermoFisher	1:500 (IF)
rb‐α‐TGN46	Abcam	1:500 (IF)
ms‐α‐GFP (Living Colors®)	TakaraBio	1:1000 (WB)
rb‐α‐α‐Tubulin	CellSignaling	1:1000 (WB)
rb‐α‐β‐Actin	Sigma	1:1000 (WB)
rb‐α‐VPS51	Sigma	1:500 (WB)
rb‐α‐EHD1	Abcam	1:500 (WB)
rb‐α‐HPS6	Proteintech	1:500 (WB)
ms‐α‐C5b9	DAKO	1:10 (IF)
rb‐α‐PI4K2A	Volker Haucke^[^ ^70^ ^]^	1:500 (WB)
rb‐α‐PI4K2B	Volker Haucke^[^ ^70^ ^]^	1:500 (WB)
rb‐α‐VIPAS39	Proteintech	1:500 (WB)
rb‐α‐AP3B1	Proteintech	1:500 (WB)
rb‐α‐GDPD5	Atlas Antibodies (HPA066762)	1:50 (IF), 1:20 (EM)
ch‐α‐GDE2/GDPD5	Shanthini Sockanathan^[^ ^25^ ^]^	1:500 (WB)
rb‐α‐Rab5	Cell Signaling	1:200 (IF)
rb‐α‐p65	Cell Signaling	1:250 (IF)
rb‐α‐Rab3B	Proteintech	1:500 (WB)

### Flow Cytometry Assay for Cell Surface GPI‐AP

HUVEC were transfected with either mCerulean‐C1, mCerulean‐C1‐GDPD5 or mCerulean‐C1‐GPDPD H233A and CD59‐FLAG, NT5E‐FLAG or TFPI‐FLAG, respectively, and then cultivated for 24 h before the GPI‐AP flow cytometry assay was performed. In control experiments, PI‐PLC treatment (0.1 U mL^−1^) was started 1 h before the actual assay. Cells were washed with PBS++ and subsequently detached from the dish using accutase (Merck). After an additional washing step, cells were fixed for 10 min with 4% PFA in PBS. Fixed cells were then blocked in 5% BSA (+1 mM CaCl_2_*2H_2_O) in PBS for 15 min. For staining of the FLAG tag, a self‐conjugated mouse‐anti‐FLAG(M1)‐DL488 antibody was applied for 1 h at room temperature (RT) in the dark. Cells were then washed 3 times and subsequently analyzed on a Guava easyCyte 11 Benchtop Flow Cytometer. Per condition, 10 000 mCerulean positive cells were counted and analyzed for their surface anti‐FLAG‐DL488 signal. In knockdown experiments, HUVEC were analyzed 24 h after the second transfection with siRNA. Per condition 20 000 HUVEC were counted and analyzed for their surface anti‐FLAG‐DL488 signal.

Surface level of endogenous CD59 was as follows: Cells were not fixed and blocked with 5% BSA in PBS for 30 min at 4 °C. Cells were then incubated with CD59‐FITC antibody for 1 h at 4 °C, washed and subsequently analyzed for the surface FITC signal.

For the FLAER‐AF488 staining assay, HUVEC were treated as described above with minor changes. HUVEC were transfected with either mCerulean‐C1, mCerulean‐C1‐GDPD5 or mCerulean‐C1‐GPDPD H233A and then cultivated for 24 h before the assay was performed. Staining of the cells with FLAER‐AF488 was performed for 30 min at room temperature in the dark.

### Electron Microscopy – Tokuyasu‐Immuno Gold Labelling on Cryosections

HUVEC were fixed in 2% paraformaldehyde, 0.2% glutaraldehyde in 0.1 M PHEM buffer, pH 7.2. Samples were processed further for cryosectioning and immuno gold labelling as described.^[^ ^73^ ^]^ For double immunogold labelling of ultrathin cryosections the GDPD5 protein was marked with the rabbit antibody, diluted 1:20 in the first step, labelled by 10 nm protein A gold (CMC, Utrecht, the Netherlands). After stabilizing the components with 1% glutaraldehyde the second labelling step was directed against VWF, using the rabbit antibody diluted 1:100 and subsequently decorated with 15 nm protein A gold. Sections were analyzed at a transmission electron microscope, operated at 80 kV (Tecani12‐Biotwin, Thermo Fisher Scientific). Representative images were acquired on ditabis imaging plates (Ditabis, Pforzheim, Germany) and on a 2 K CCD camera (Veleta, EMSIS, Münster, Germany).

### Antibody Uptake Assay

GDPD5‐3xHA‐EGFP carrying a triple HA tag in the first extracellular loop was ectopically expressed in HUVEC. 8 h post transfection, a monoclonal anti‐HA tag antibody was added to the medium, and the cells were kept for another 8–24 h, washed and then fixed and permeabilized for subsequent immunofluorescence staining.

### Immunofluorescence Staining

HUVEC were seeded on collagen‐coated 12 mm glass cover slips and cultivated at 37 °C, 5% CO_2_. Cells were fixed in 4 % PFA in 1x PBS for 10 min at RT and subsequently permeabilized using 0.1 % Triton X‐100 for 2 min. For methanol fixation, cells were fixed and permeabilized in methanol for 5 min at −20 °C. After blocking (3% BSA in 1x PBS) for 30 min, primary antibodies were applied at the dilutions indicated. Cells were washed and treated with secondary antibodies and DAPI, if applicable. After final washing, HUVEC were mounted on glass slides using Mowiol. Samples were analyzed using a confocal laser scanning microscope, LSM800 (Zeiss).

### Live Cell Microscopy

HUVEC transfected with the appropriate constructs were seeded and imaged in collagen‐coated 8‐well glass bottom µ‐slides (ibidi). During acquisition, cells were maintained in mixed endothelial growth medium containing 20 mM HEPES at 37 °C. Histamine stimulation was carried out manually during acquisition by adding histamine stock solution (diluted in Mix‐Medium + 20 mM HEPES) to a final concentration of 500 µM histamine. VEGF/ATP stimulation was carried out manually during acquisition by adding VEGF/ATP mix to a final concentration of 50 ng*mL^−1^/100 µM VEGF/ATP. Confocal microscopy was performed on a LSM780 confocal system (Zeiss) using a 63×, NA 1.4, oil immersion objective (Plan‐Apochromat, Zeiss).

### Colocalization Analysis

Analysis of microscopic images was performed using Fiji software.^[^ ^74^ ^]^ For colocalization analyses, z‐stacked images of HUVEC, which expressed EGFP‐tagged GDPD5 constructs and were stained with the different antibodies, were quantified for their Pearson correlation coefficient with the indicated protein/organelle ratio using the JaCoP‐Plugin.^[^ ^75^ ^]^ Additionally Cell Profiler (Version 4.2.5) was used together with the Organelle Content pipeline to analyze colocalization between EGFP‐GDPD5 and VWF.^[^ ^34^ ^]^ HUVEC expressing EGFP‐tagged GDPD5 were cut out via Fiji before analysis.

### CD59 ELISA

HUVEC were transfected with GDPD5‐EGFP or left untransfected and seeded onto 6 cm plates. 32 h later, cells were washed and incubated with starvation medium (M199 supplemented with 2%BSA, 30 µg mL^−1^ gentamycin and 0.015 µg mL^−1^ amphotericin B) for 1 h. Supernatants were collected, and cells were incubated with starvation medium, supplemented with 500 µM histamine for 1 h. Supernatants were again collected and analyzed using the CD59‐ELISA Kit (Thermo Fisher, # EH76RB) according to the manufacturer's instructions.

### MAC Assay

HUVEC were transfected with GDPD5‐EGFP and seeded onto glass coverslips in 24‐well plates. 28 h post transfection, medium was replaced by medium containing 20% human serum. 3 h later, histamine (500 µM) was added to half of the wells and cells were cultured for 1 h. Cells were then fixed with 4% PFA in 1x PBS for 10 min at RT and the normal IF staining protocol was performed, skipping the Triton‐X100 permeabilization.

### NFκB‐Translocation Assay

HUVEC were transfected with EGFP‐GDPD5 or EGFP and seeded onto glass coverslips in 24‐well plates. 24 h post transfection, medium was replaced by medium containing 30% human serum supplemented with/without 500 µM histamine. 2 h later cells were fixed with 4% PFA in 1x PBS for 10 min at RT and the normal IF staining protocol was performed.

### Statistics

All statistical analyses were performed using GraphPad Prism. Asterisks mark statistically significant results: ****p ≤ 0.0001, ***p ≤ 0.001, **p ≤ 0.01, *p ≤ 0.05, ns = not significant. Normal distribution was assessed by the Shapiro‐Wilk‐Test (n ≤ 8) or D'Agostino‐Pearson test (n > 8), p < 0.05. Normally distributed data were analyzed employing Student´s t‐test or one‐way Anova with Dunnett's post hoc test. Non‐parametric data was analyzed using Mann‐Whitney test, paired Wilcoxon test or Kruskal Wallis with Dunn's post hoc test, as detailed in the respective figure legends.

## Conflict of Interest

The authors declare no conflict of interest.

## Author Contributions

J.N. and V.G. performed conceptualization; J.N., J.T., D.Z., and V.G. performed methodology; J.N., J.T., and D.Z. performed investigation; J.N., J.T., D.Z., and V.G. performed visualization; V.G. performed supervision; J.N., D.Z., and V.G. wrote the original draft and also reviewed and edited.

## Supporting information

Supporting Information

## Data Availability

The data that support the findings of this study are available from the corresponding author upon reasonable request.
