# Supplementary material for: Evoked Weibel‐Palade Body Exocytosis Modifies the Endothelial Cell Surface by Releasing a Substrate‐Selective Phosphodiesterase
Source: Adv Sci (Weinh). 2024 Feb 15;11(16):2306624. doi: 10.1002/advs.202306624 (PMC11040351; doi:10.1002/advs.202306624)
Supplement: Supplementary file 1 — Supporting Information [file ADVS-11-2306624-s001.pdf]

ADVANCED SCIENCE
Open Access

Supporting Information

for *Adv. Sci.*, DOI 10.1002/advs.202306624

Evoked Weibel-Palade Body Exocytosis Modifies the Endothelial Cell Surface by Releasing a Substrate-Selective Phosphodiesterase

*Johannes Naß, Julian Terglane, Dagmar Zeuschner and Volker Gerke**

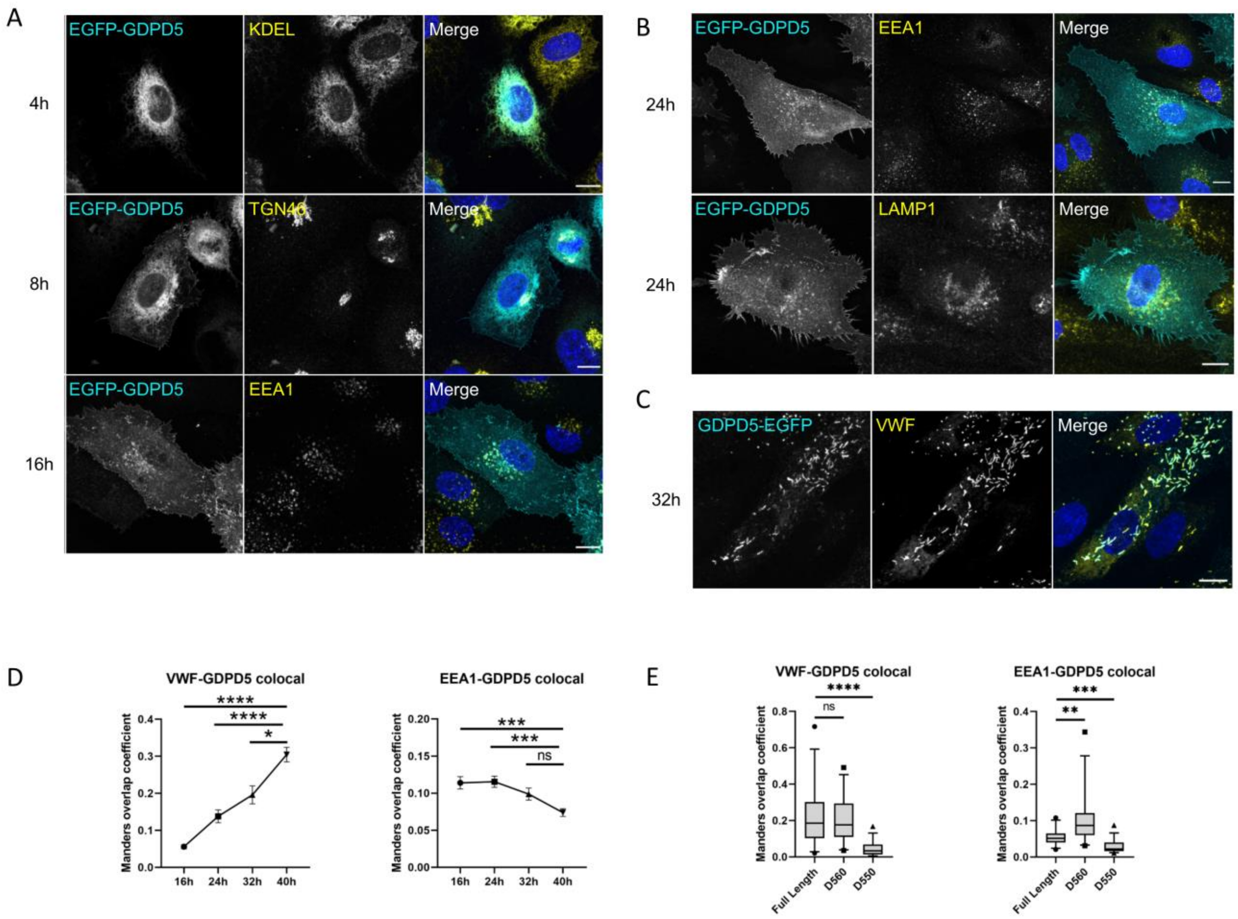

Figure S1. Localization of GDPD5 in HUVEC. A, B: HUVEC were transfected with EGFP-GDPD5 and fixed at the indicated time points after transfection. Following permeabilization, cells were stained for KDEL as an ER marker, TGN46 as a trans-Golgi network marker and EEA1 as an early endosome marker (**A**) or EEA1 and LAMP1 as late endosome marker (**B**). and subjected to confocal laser scanning microscopy. Scale bar: 10 µm. **C:** HUVEC were transfected with GDPD5-EGFP, fixed after 32h, stained for VWF and analyzed by confocal microscopy. Scale bar: 10 µm. Note that the C-terminally EGFP-tagged GDPD5 (**C**) construct shows the same localization as the N-terminally tagged EGFP-GDPD5 (**A**, Fig 1). **D:** Colocalization between EGFP-GDPD5 and the markers indicated was analyzed using Manders overlap coefficient. Error bars=SEM, $n \geq 26$ cells from 3 different experiments. Significance was tested using Kruskal Wallis with Dunn's test for multiple comparisons. (* $p < 0.05$, *** $p < 0.001$, **** $p < 0.0001$, ns = not significant)**. E:** Colocalization between the different EGFP-tagged GDPD5 constructs and the indicated marker was analyzed using Manders overlap coefficient (GDPD5 signal on VWF/EEA1 signal). $n \geq 31$ cells from 3 different experiments. Significance was tested using ordinary Kruskal

Wallis with Dunn's test for multiple comparisons. Box plots with 5-95% confidence interval. (* $p < 0.05$, ** $p < 0.01$, *** $p < 0.001$, **** $p < 0.0001$, ns = not significant).

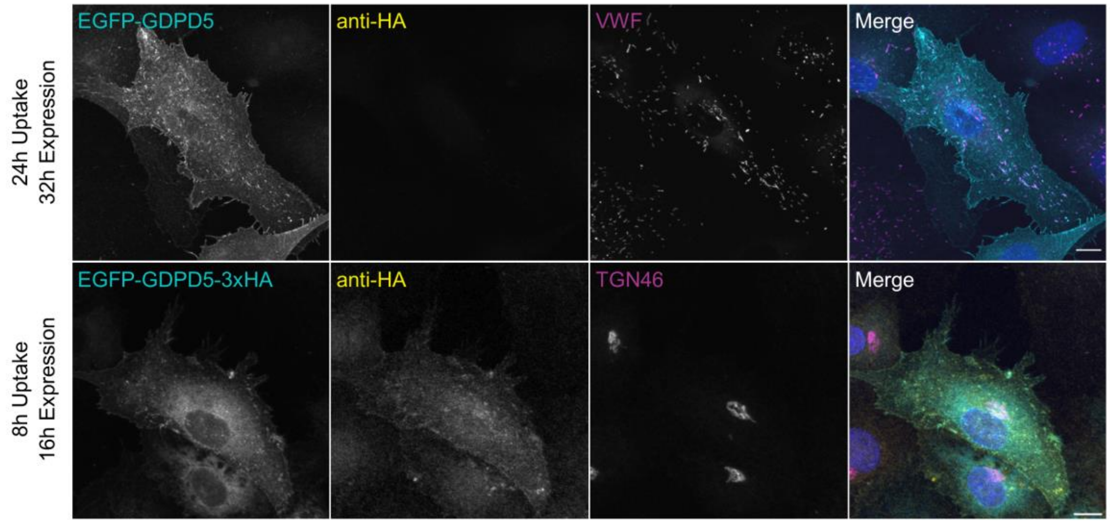

Figure S2. Antibody Uptake Assay requires extracellular HA epitope and does not pass the TGN. GDPD5-EGFP (upper panel) or GDPD5-3xHA-EGFP (lower panel) carrying a triple HA tag in the first extracellular loop was ectopically expressed in HUVEC. 8h post transfection, a monoclonal anti-HA tag antibody was added to the medium, and the cells were kept for another 8h-24h, washed and then fixed and permeabilized for subsequent immunofluorescence staining. EGFP signal is shown in teal, HA-antibody (uptake) in yellow, VWF (upper panel) or TGN46 (lower panel) in magenta and DAPI signal in blue. Scale bar = 10 µm.

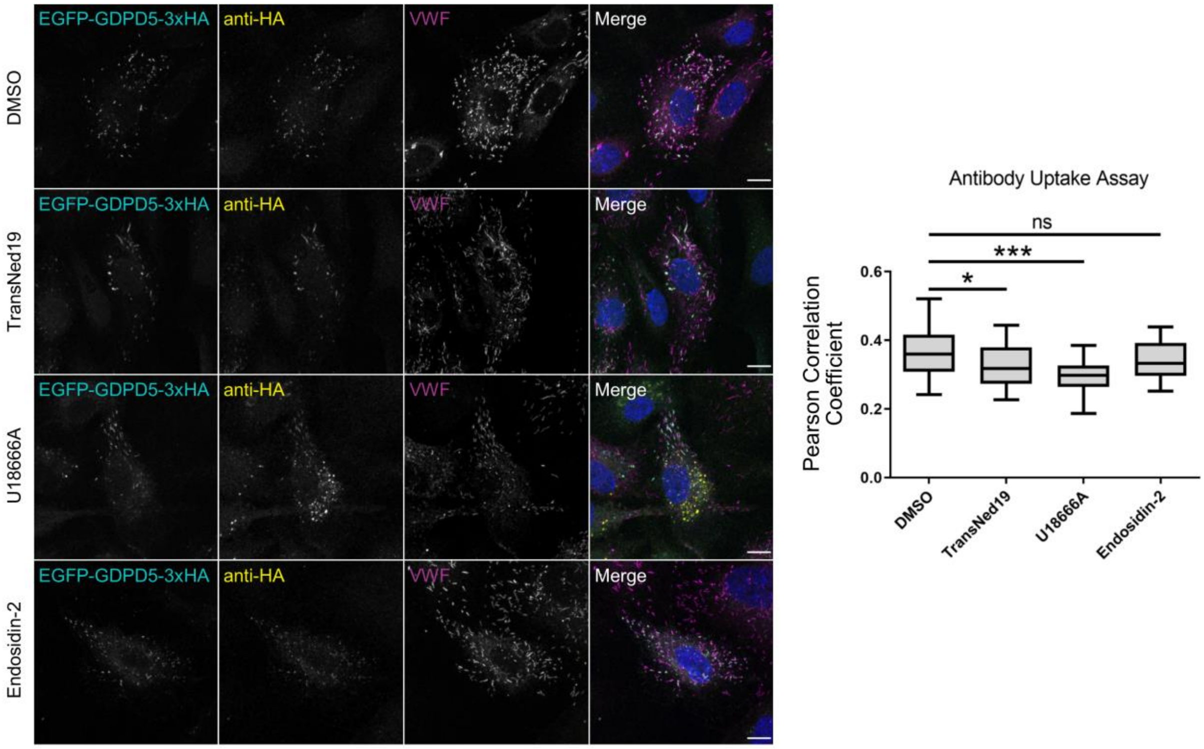

Figure S3. Different small molecule inhibitors were tested for their effect on the GDPD5 transport to WPB using the antibody uptake assay. GDPD5-3xHA-EGFP carrying a triple HA tag in the first extracellular loop was ectopically expressed in HUVEC. 8h post transfection, a monoclonal anti-HA tag antibody and the inhibitors indicated were added to the medium, and the cells were kept for another 24h, washed and then fixed and permeabilized for subsequent immunofluorescence staining. Examples of representative images of fixed samples also stained with anti-VWF antibodies (magenta) are shown on the left. EGFP signal is shown in teal, HA-antibody (uptake) in yellow and DAPI signal in blue. Scale bar = 10 µm. Colocalization between the anti-HA tag antibody and the anti-VWF antibody signal was analyzed using Pearson correlation coefficient. n ≥ 24 cells from 3 different experiments (right). Significance was tested using ordinary one-way ANOVA with Dunnetts test for multiple comparisons. (* $p < 0.05$, *** $p < 0.001$, ns = not significant). Note that transport of the anti-HA tag antibodies to WPB is not affected by any of the treatments; the significant reduction in Pearson correlation coefficient seen in particular following U18666A treatment is most likely due to an accumulation of antibody internalized independently of the GDPD5-3xHA-EGFP into perinuclear structures probably reflecting lysosomes/late endosomes that are enlarged due to the NPC1 inhibition by U18666A.

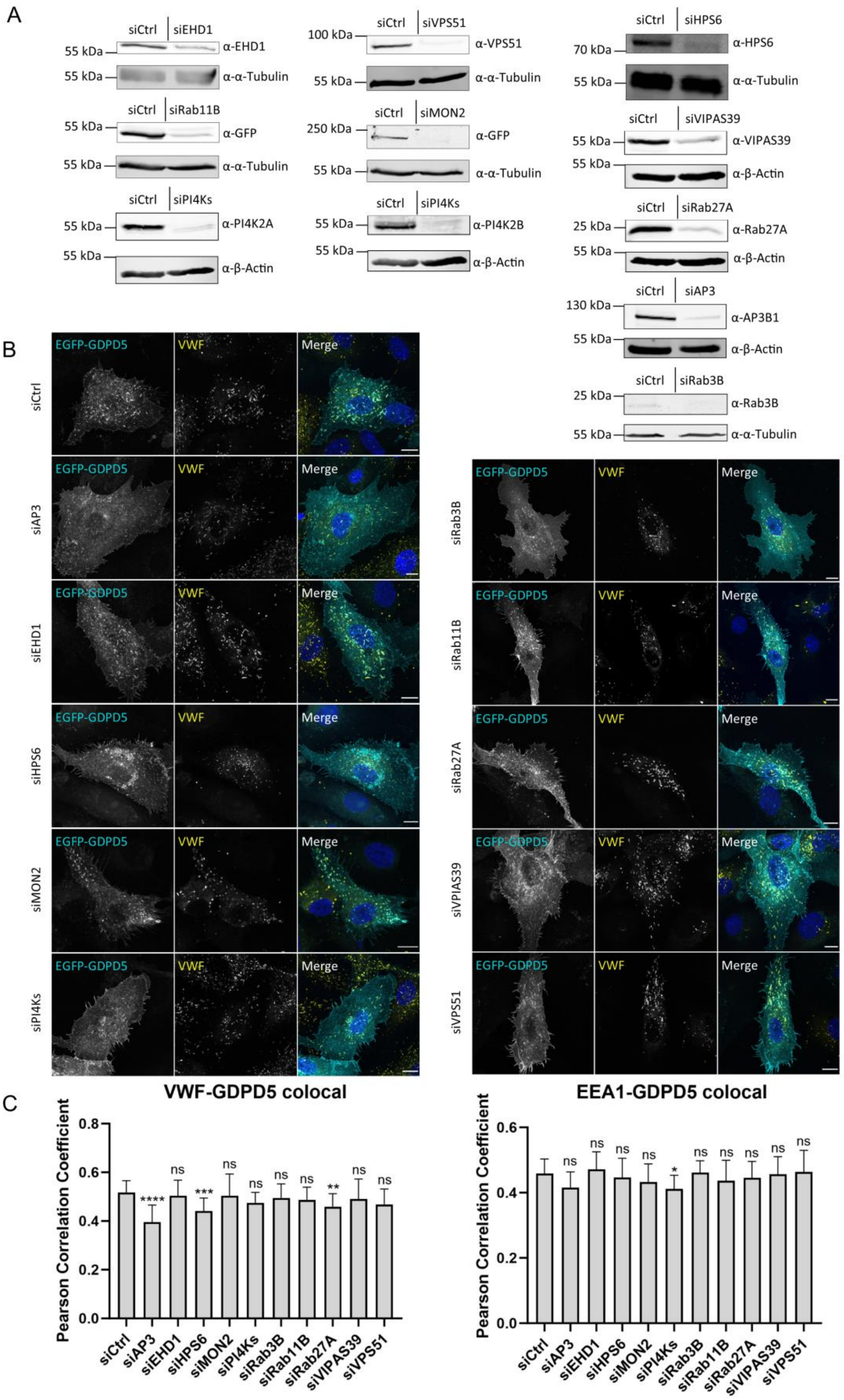

A
siCtrl
siEHD1
α-EHD1
α-α-Tubulin
siRab11B
α-GFP
siPI4Ks
α-PI4K2A
α-β-Actin
siVPS51
α-VPS51
siMON2
α-PI4K2B
siHPS6
α-HPS6
siVIPAS39
α-VIPAS39
siRab27A
α-Rab27A
siAP3
α-AP3B1
siRab3B
α-Rab3B
B
EGFP-GDPD5
VWF
Merge
C
VWF-GDPD5 colocal
EEA1-GDPD5 colocal
Pearson Correlation Coefficient

Figure S4. Knockdown of endosomal transport proteins and GDPD5 localization. A: HUVEC were transfected with the respective siRNA (500 pmol siVIPAS39, 300 pmol each siPI4Ks, 400 pmol each other siRNA), kept for 48h and then again transfected with the respective siRNA. In case of Rab11B and MON2, due to the lack of appropriate primary antibodies, cells were cotransfected with a respective EGFP-tagged construct to assess knockdown efficiency by anti-GFP antibody blot. Lysates were prepared 32 h after the second transfection and analyzed via Western blot employing the primary antibodies indicated. **B:** HUVEC were transfected with the respective siRNA (500 pmol siVIPAS39, 300 pmol each siPI4Ks, 400 pmol each other siRNA), kept for 48h, again transfected with the respective siRNA plus GDPD5-EGFP and fixed 32h after the second transfection. Cells were then processed for immunofluorescence and stained for VWF as a WPB marker. Representative images of the colocalization of GDPD5-EGFP and VWF are shown (teal = GDPD5-EGFP, yellow = VWF, blue = nucleus/DAPI). Scale bar = 10 µm. **C:** Colocalization between EGFP-GDPD5 and the indicated markers was analyzed using Pearson correlation coefficient. n = 32 cells from 4 different experiments for each condition or n = 24 cells from 3 different experiments for Rab3B knockdown. Significance was tested using ordinary one-way ANOVA with Dunnetts test for multiple comparisons (VWF) or Kruskal Wallis test with Dunn's test for multiple comparisons (EEA1) using siCtrl as control value (* $p < 0.05$, ** $p < 0.01$, *** $p < 0.001$, **** $p < 0.0001$, ns = not significant). Shown are bar graphs. Error bars = SD.

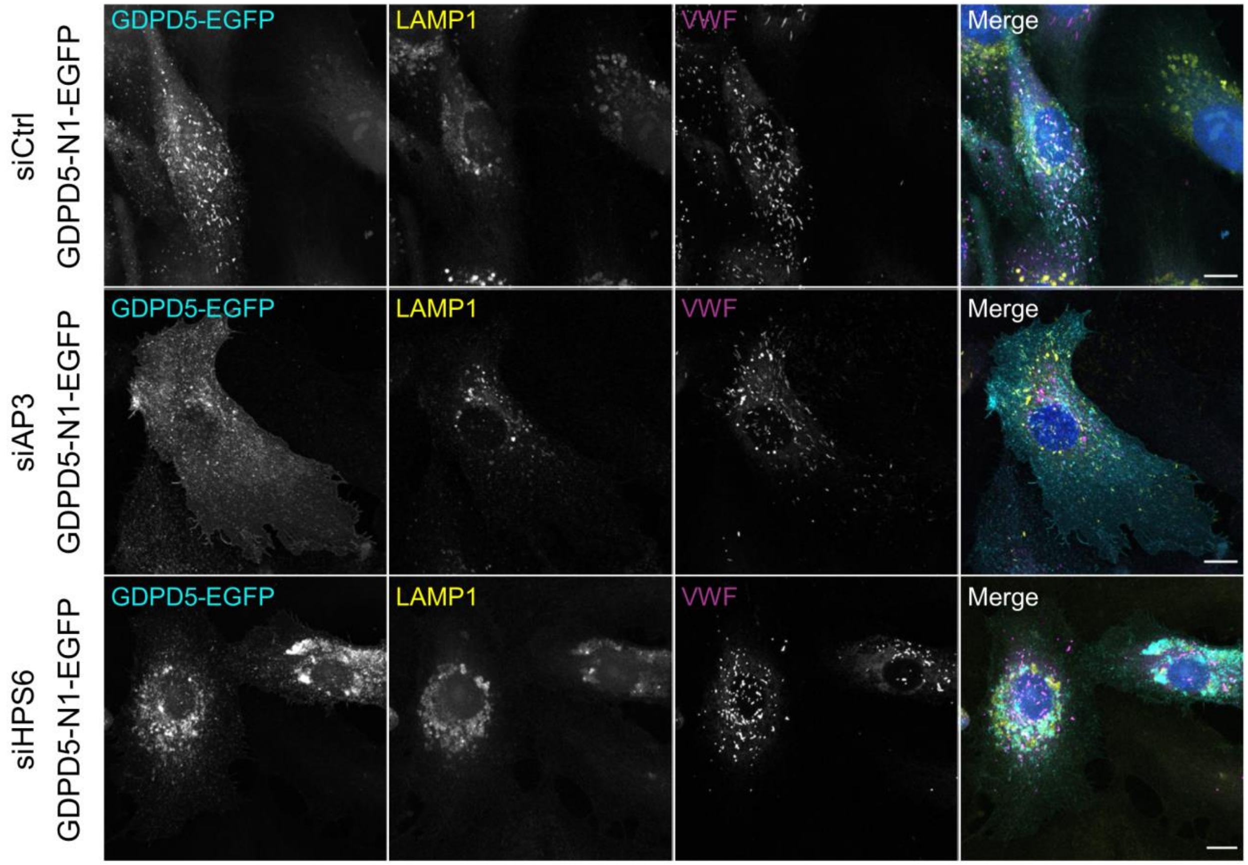

Figure S5. HPS6 and AP3 knockdown affects the localization of GDPD5. HUVEC were transfected with 400 pmol of the respective siRNA, kept for 48h, transfected again with 400 pmol siRNA and GDPD5-N1-EGFP (EGFP tagged to C-terminal end of GDPD5), and kept for 32h. Cells were then subjected to immunofluorescence and stained for LAMP1 as late endosomal marker and VWF as WPB marker. Scale bar: 10 µm.

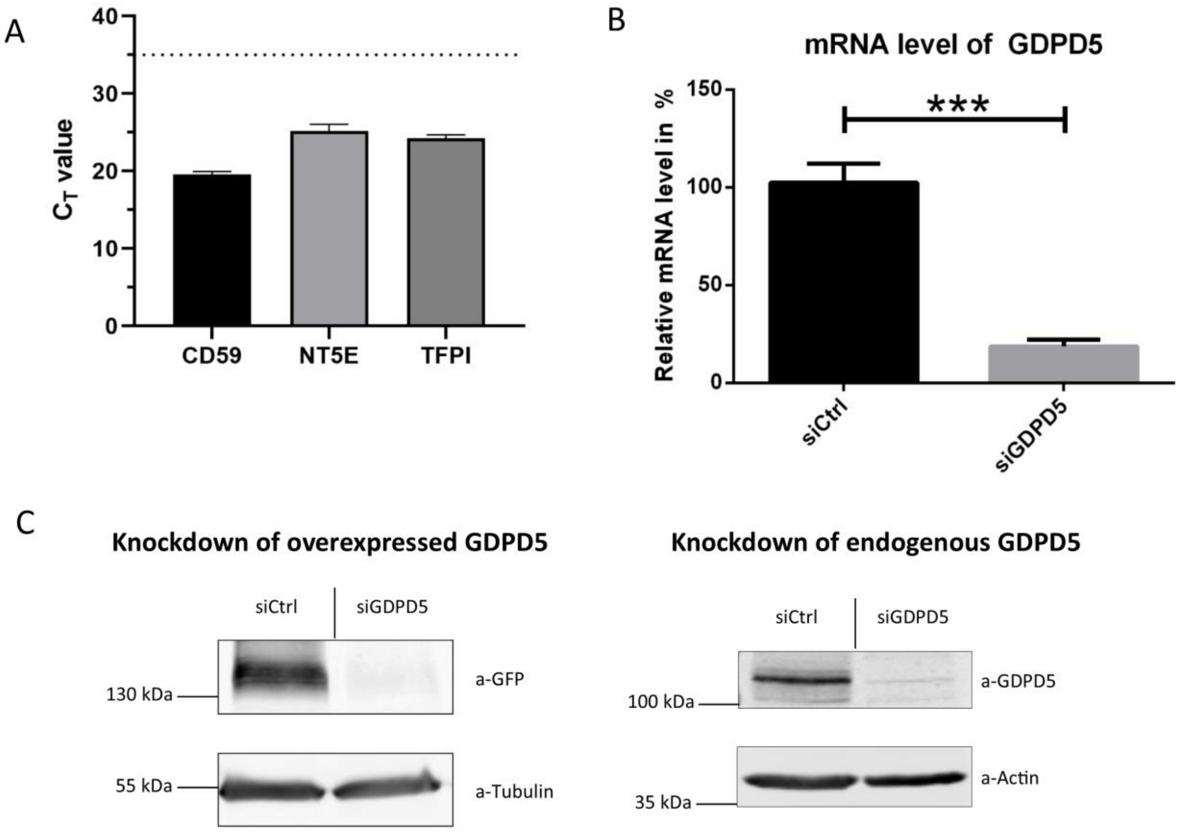

Figure S6. mRNA and protein level of certain GPI-AP and GDPD5 in HUVEC. A: The mRNA expression levels of CD59, NT5E and TFPI were analyzed by RT-PCR. The threshold cycle value (C_T) considered relevant for protein expression was set to 35 (dotted line). Data represent means from n=4 independent experiments. Error bars = SD. **B.** HUVEC were transfected with 200 pmol of siGDPD5 or siCtrl for 48 h, and then transfected again with the same amounts of the respective siRNAs. RNA was isolated 24h after the second transfection and analyzed via RT-PCR. N=6 experiments, significance tested with unpaired student's t-test (*** $p < 0.001$). Error bars = SEM. **C.** siRNA mediated knockdown of ectopically expressed EGFP-GDPD5 (left) and endogenous GDPD5 (right). HUVEC were transfected with 200 pmol of siGDPD5 or siCtrl for 48 h, and then transfected again with the same amounts of the respective siRNAs and EGFP-GDPD5. Lysates were prepared 24 h after the second transfection and analyzed via Western blot using anti-GFP antibodies. For endogenous knockdown HUVEC were only transfected with siRNA for the second round of transfection. Anti-GDPD5 antibodies were used for analysis.

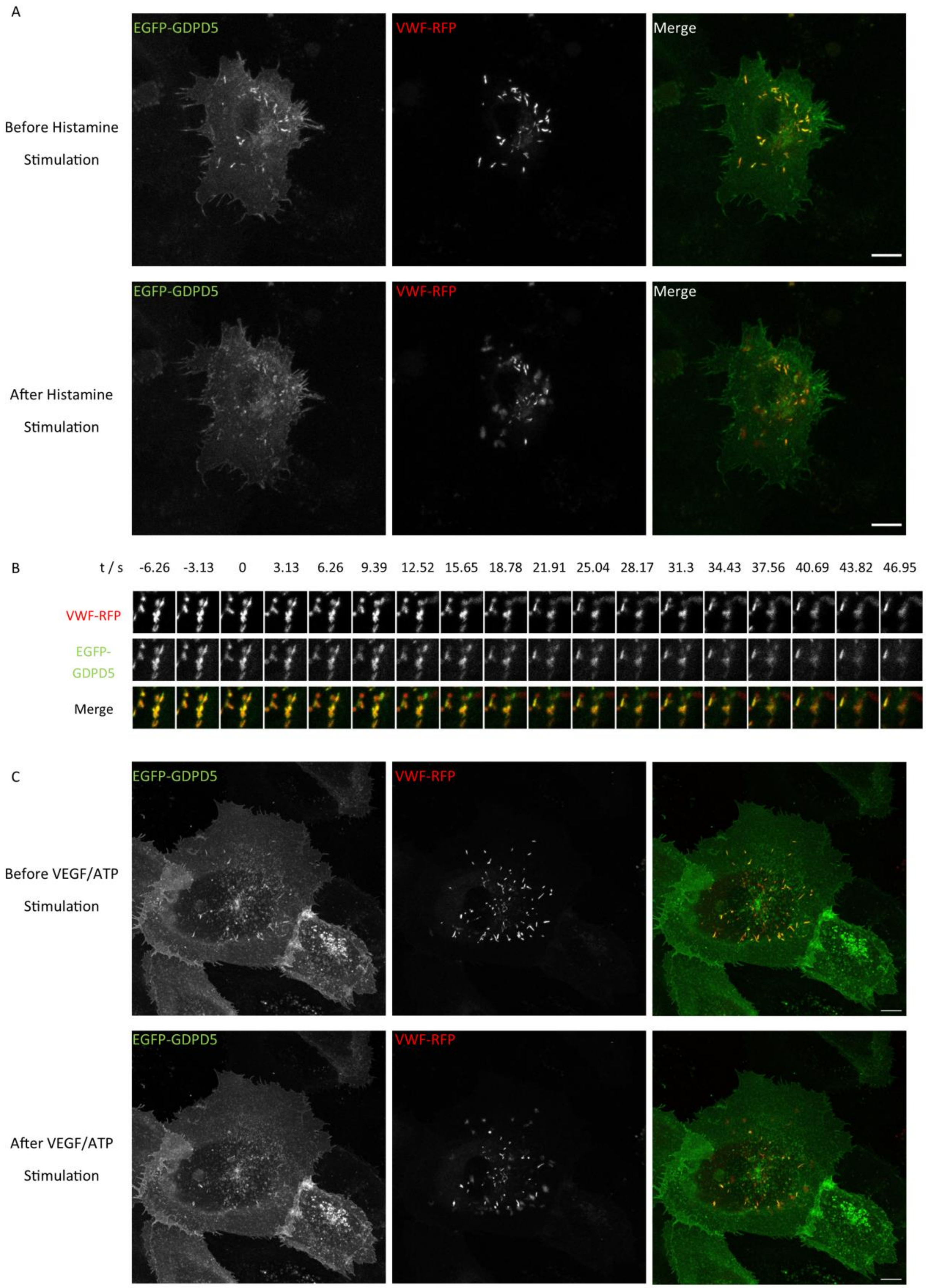
A
EGFP-GDPD5
VWF-RFP
Merge
Before Histamine Stimulation
EGFP-GDPD5
VWF-RFP
Merge
After Histamine Stimulation
B
t / s
-6.26
-3.13
0
3.13
6.26
9.39
12.52
15.65
18.78
21.91
25.04
28.17
31.3
34.43
37.56
40.69
43.82
46.95
VWF-RFP
EGFP-GDPD5
Merge
C
EGFP-GDPD5
VWF-RFP
Before VEGF/ATP Stimulation
EGFP-GDPD5
VWF-RFP
After VEGF/ATP Stimulation

Figure S7. GDPD5 positive WPB exocytose after stimulation. HUVEC ectopically expressing EGFP-GDPD5 and VWF-RFP were subjected to time-lapse confocal microscopy and stimulated with 500 µM histamine (**A** and **B**) or 50 ng*mL^{-1}/100 µM VEGF/ATP (**C**) during acquisition. **A.** Stills of a representative movie taken before stimulation and after few WPB had already undergone fusion (after histamine stimulation). Scale bar: 10 µm. **B**. Higher magnification stills of a few WPB undergoing fusion. Note the decrease in EGFP-GDPD5 signal following exocytosis. Time in seconds is given with respect to histamine addition at t=0. **C.** Stills of a representative movie taken before stimulation and after few WPB had already undergone fusion (after VEGF/ATP stimulation). Scale bar: 10 µm.

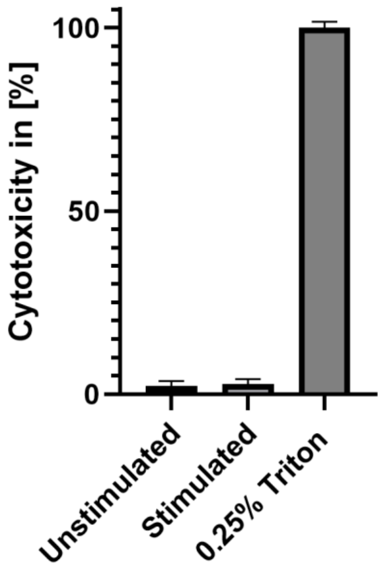

Figure S8. Enhanced MAC deposition after GDPD5 release does not cause cell lysis. HUVEC were transfected with GDPD5-EGFP and then cultured for 32h. Cells were then treated with 20% human serum for 4h and either left unstimulated or stimulated for 1h with histamine (500 µM). In control experiments, cells were treated for 30 min with 0.25% Triton (positive control). Subsequently, cell culture supernatants were analyzed with a LDH Cytotoxicity Kit for LDH release. n = 3 experiments. Error bars = SEM.

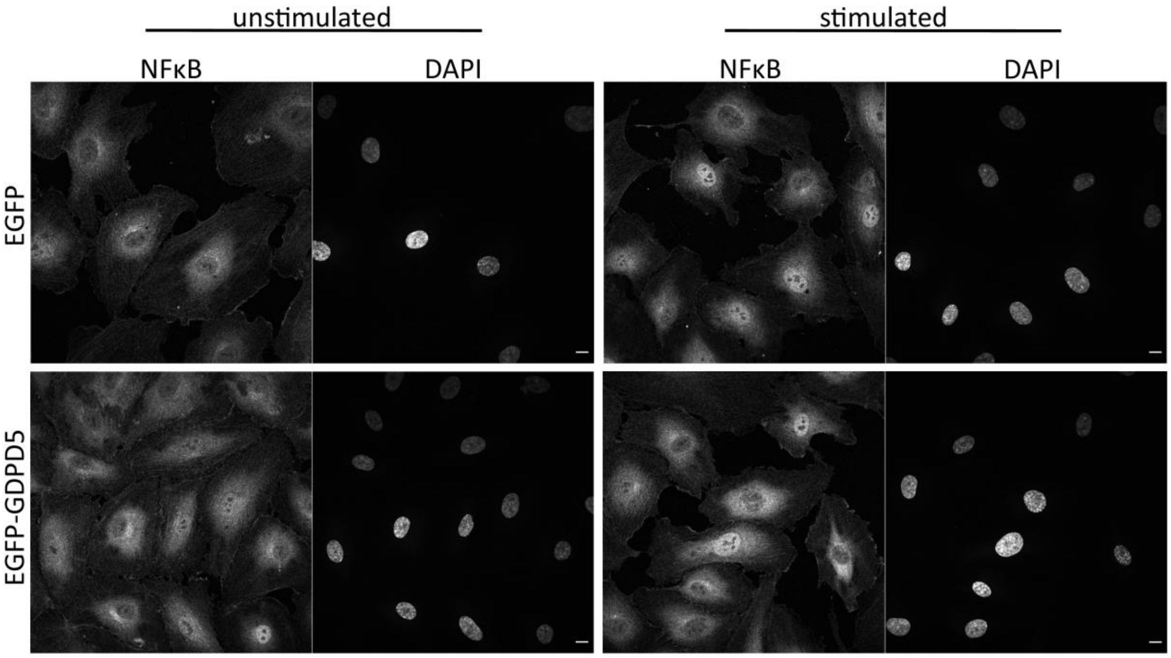

Figure S9. Histamine stimulation of HUVEC treated with human serum results in nuclear translocation of NFκB. HUVEC ectopically expressing EGFP or EGFP-GDPD5 were treated with 30 % human serum supplemented with or without 500 µM histamine for 2 h and subsequently subjected to immunostaining using anti-p65 antibodies. Shown are images of maximum intensity projections of z-stacks. Scale bars = 10 µm.

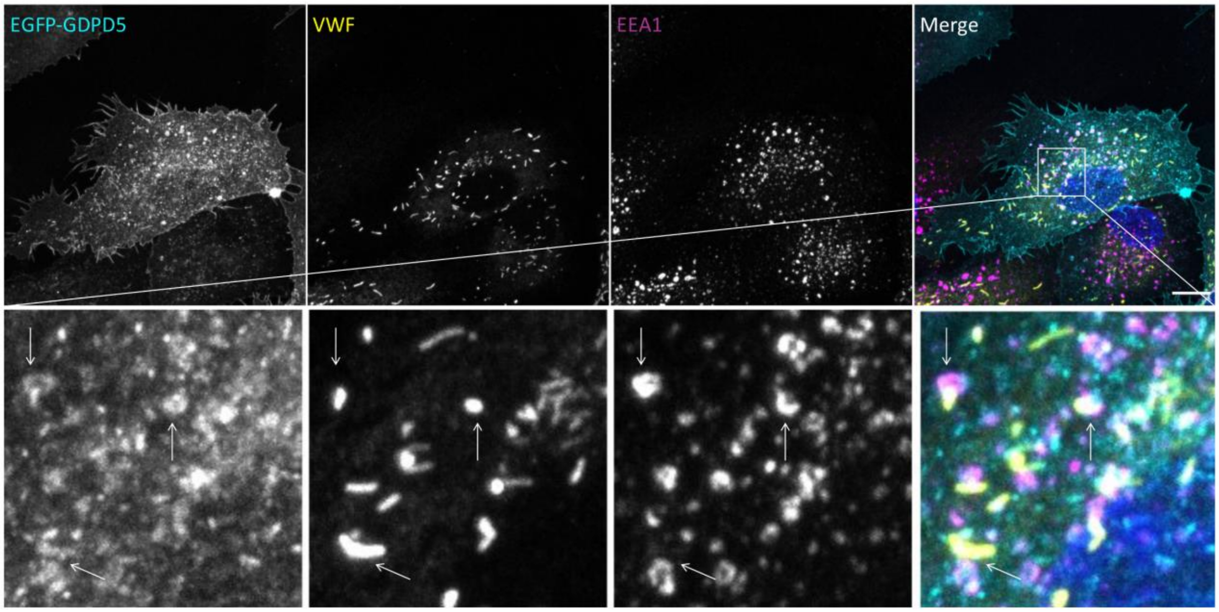

Figure S10. GDPD5 positive EE and WPB reside in close proximity. HUVEC ectopically expressing GDPD5-EGFP were fixed 24h post transfection and then subjected to immunofluorescence staining for VWF as WPB marker and EEA1 as an early endosome marker. Arrows highlight endosomes positive for EEA1 and GDPD5 that are in close proximity to WPB. Scale bar = 10 µm.
